# Genome characterization of the selected long- and short-sleep mouse lines

**DOI:** 10.1007/s00335-016-9663-6

**Published:** 2016-09-20

**Authors:** Robin Dowell, Aaron Odell, Phillip Richmond, Daniel Malmer, Eitan Halper-Stromberg, Beth Bennett, Colin Larson, Sonia Leach, Richard A. Radcliffe

**Affiliations:** 1 BioFrontiers Institute, University of Colorado Boulder, Boulder, CO 80309 USA; 2 Department of Molecular, Cellular, and Developmental Biology, University of Colorado Boulder, Boulder, CO 80309 USA; 3 Department of Computer Science, University of Colorado Boulder, Boulder, CO 80309 USA; 4 Center for Genes, Environment and Health, National Jewish Health, Denver, CO 80206 USA; 5 Skaggs School of Pharmacy and Pharmaceutical Sciences, University of Colorado, Aurora, CO 80045 USA

## Abstract

The Inbred Long- and Short-Sleep (ILS, ISS) mouse lines were selected for differences in acute ethanol sensitivity using the loss of righting response (LORR) as the selection trait. The lines show an over tenfold difference in LORR and, along with a recombinant inbred panel derived from them (the LXS), have been widely used to dissect the genetic underpinnings of acute ethanol sensitivity. Here we have sequenced the genomes of the ILS and ISS to investigate the DNA variants that contribute to their sensitivity difference. We identified ~2.7 million high-confidence SNPs and small indels and ~7000 structural variants between the lines; variants were found to occur in 6382 annotated genes. Using a hidden Markov model, we were able to reconstruct the genome-wide ancestry patterns of the eight inbred progenitor strains from which the ILS and ISS were derived, and found that quantitative trait loci that have been mapped for LORR were slightly enriched for DNA variants. Finally, by mapping and quantifying RNA-seq reads from the ILS and ISS to their strain-specific genomes rather than to the reference genome, we found a substantial improvement in a differential expression analysis between the lines. This work will help in identifying and characterizing the DNA sequence variants that contribute to the difference in ethanol sensitivity between the ILS and ISS and will also aid in accurate quantification of RNA-seq data generated from the LXS RIs.

**Electronic supplementary material:**

The online version of this article (doi:10.1007/s00335-016-9663-6) contains supplementary material, which is available to authorized users.

## Introduction

McClearn (1962) observed that inbred laboratory mouse strains differed substantially in the duration of the loss of righting response, or “sleep time,” following an acute high dose of alcohol (ethanol). He also noted that sleep time showed an inverse correlation with a strain’s preference for drinking an alcohol solution over water, which is consistent with the well-supported observation in humans that individuals who are less sensitive to the sedative effects of alcohol are at greater risk for developing alcohol-related problems (Schuckit 1994), although it has been argued that increased sensitivity to alcohol’s stimulatory effects is also a contributing factor (King et al. 2014). It was subsequently found that the difference in sleep time was due primarily to brain sensitivity and not to differences in alcohol metabolism (Kakihana et al. 1966). Since McClearn’s original observation, the sleep time assay has become perhaps the most widely used test of acute alcohol sensitivity in model organisms, yet its genetic underpinnings remain elusive.

McClearn and Kakihana (1981) undertook a bidirectional selection experiment to create lines of mice that would differ in their sleep time postulating that they would be useful for investigating the genetics of this response and ultimately learn how acute sensitivity relates to drinking behavior. The founder population was a “heterogeneous stock” (HS) which was derived from eight inbred strains and maintained through restricted random mating (McClearn et al. 1970). Sleep time was measured by injecting the mice with 3.3 g/kg alcohol and measuring the time from the loss to the regain of the righting response. The dose was increased several times during selection because the Short-Sleep (SS) line failed to respond as it accumulated low-sensitivity alleles. After five generations, the difference in sleep time between the Long-Sleep (LS) and SS lines was approximately threefold and over tenfold difference after 18 generations. Nearly 40 years later, the ancestors of those lines, now inbred and referred to as the ILS and ISS, still maintain their extreme difference in acute alcohol sensitivity with the ISS requiring roughly twice the amount of alcohol than the ILS for the two strains to achieve similar sleep times (Radcliffe et al. 2006).

Recognizing that a single pair of selected lines had limited utility for genetic studies, a recombinant inbred (RI) panel was derived from the outbred LS and SS (DeFries et al. 1989); this panel no longer exists. A second RI panel, known as the LXS, was created from the ILS and ISS and currently consists of over 60 strains (Williams et al. 2004). The LXS panel was created from pairs of ILS/ISS-derived F2 offspring that were bred through brother–sister matings for more than 20 generations resulting in a panel of inbred strains, each of which contains a random assortment of alleles from the ILS and ISS (Williams et al., 2004). RI panels have been invaluable for a variety of complex traits analysis approaches, including genetic correlation analysis and quantitative trait locus (QTL) mapping (Gora-Maslak et al. 1991). More recently, both of these approaches have been combined with massively parallel, high-throughput gene expression analysis in what has been referred to as “genetical genomics” which aids in the identification of specific genes that may contribute to genetic variation for a trait of interest (Chesler et al. 2005; Jansen and Nap 2001).

We found that the sleep time difference in the ILS and ISS appeared to be resulting from a substantial difference in acute functional tolerance (AFT), at least in part (Radcliffe et al. 2006). AFT, first noted by Mellanby (1919), is the development of alcohol tolerance within a drinking session and it is thought to be a critical factor in the relationship between acute sensitivity and alcoholism risk noted above, although this relationship has not been firmly established (Bujarski et al. 2015; Fillmore and Weafer 2012; King et al. 2014; Newlin and Thomson 1990). More recently, with the use of the LXS RI panel, we showed a highly significant genetic correlation between AFT and drinking behavior and also mapped a significant QTL for AFT on distal chromosome 4 where others have also mapped QTLs for drinking behavior (Belknap and Atkins 2001; Bennett et al. 2015; Radcliffe et al. 2013; Saba et al. 2011). In addition to our AFT mapping study, sleep time has been mapped in the LXS (Bennett et al. 2006) and they also have been used for genetic analysis of a wide variety of alcohol and non-alcohol-related traits such as low-dose alcohol activation (Downing et al. 2006), alcohol drinking (Saba et al. 2011), hearing loss (Noben-Trauth et al. 2010), dietary restriction-mediated lifespan (Rikke et al. 2010), and body weight (Bennett et al. 2005).

The QTL approach has not been as fruitful as first envisioned, i.e., very few “QT genes” have been identified despite the many thousands of QTLs that have been mapped (Flint et al. 2005). However, technological and analytical advances, including high-throughput gene expression analysis and “Next-Generation” deep sequencing technologies (NextGen), are providing an unprecedented opportunity to examine the molecular basis of QTLs (Harrison 2012).

Here we report on the genome resequencing of the ILS and ISS mouse strains using Illumina short-read deep sequencing technology. Our strategy used a combination of three libraries and paired-end sequencing to generate a catalog of variants between the two strains. We have also been able to delineate the ancestral origin of the strains using a hidden Markov model approach with sequence data from six of the eight strains that went into the ILS and ISS (Keane et al. 2011); one of the original HS progenitor strains (Is/Bi) has long been lost and neither its sequence nor DNA is available and the original RII strain is no longer available, though The Jackson Laboratory carries several sub-strains that were derived from the RIII. We have examined the variants that fall into QTLs that have been mapped for the original selection trait and other similar traits (e.g., AFT). Finally, in conjunction with our quantitative RNA sequencing (RNA-seq) of the brains of the ILS and ISS strains, we demonstrate the importance of aligning RNA-seq reads to the genome from which they were generated.

## Materials and methods

### Animals

The original Long- and Short-Sleep lines were selected based on the duration of the loss of the righting response from the first generation of a “heterogeneous stock” (HS) which was created through a systematic intercrossing scheme of 8 inbred laboratory mouse strains: A, AK, BALB/c, C3H, C57BL, DBA/2, Is/Bi, and RIII (McClearn and Kakihana 1981; McClearn et al. 1970; the nomenclature for the strain names used here and throughout the rest of this paper is that used by the authors of these publications). The intercrosses were designed so as to preserve an equal frequency of the Y chromosome from each of the progenitor strains. The HS was maintained through restricted random mating.

The selected LS and SS lines were inbred in the early 1990s to create the Inbred Long- and Short-Sleep strains (ILS, ISS; Markel et al. 1997). ILS and ISS breeders were obtained from The Jackson Laboratory (Bar Harbor, ME) and bred in-house in the UCAMC vivarium, a pathogen-free facility. Offspring were weaned and sex-separated at 21 days of age. All experiments were conducted with males that were group-housed in standard housing containing 2–5 mice per cage. They were maintained in a constant temperature (22–23 °C), humidity (20–24 %), and light (14L/10D) environment. The mice were between 58 and 91 days of age (average: 74.9 ± 2.7) at the time of their use. The mice used for the RNA-seq experiment were part of a larger ongoing experiment with the ILS, ISS, and LXS RI strains examining the effects of genetics and acute alcohol on the brain transcriptome. Here we report only on control ILS and ISS mice which were sacrificed 8 h after a single intraperitoneal injection of normal saline (0.01 ml/g); mice used for genome sequencing were completely naïve. The procedures described in this report have been established to ensure the absolute highest level of humane care and use of the animals, and have been reviewed and approved by the UCAMC IACUC.

### Full genome sequencing and analysis

An overview of the ILS and ISS sequencing strategy is shown in Supplemental Figure S-1. DNA was extracted from the liver of a single male ILS and single male ISS mouse for full genome sequencing. A standard phenol/chloroform/isoamyl (PCI) procedure was used to isolate high-molecular weight DNA. Briefly, liver was dissected and flash frozen in liquid nitrogen. Following grinding by hand and tissue digestion (proteinase K), the sample was added to PCI, mixed, and centrifuged. The aqueous phase was removed and the DNA was precipitated with 7.5 M ammonium acetate. A DNA pellet was formed by centrifugation and then washed with 70 % ethanol. The pellet was dried and resuspended in 10 mM Tris (pH 7.5). RNA was removed by digestion with RNase A, followed by PCI extraction and resuspension in Tris as before.

Three short-read sequencing libraries were prepared from the DNA: a 2 × 100 paired-end library (~300 bp insert) and two 2 × 100 mate-pair libraries (~4 kb and ~10 kb insert sizes). The paired-end library was prepared using the Illumina TruSEQ DNA Library Sample Preparation Kit and the mate-pair libraries were prepared using the Illumina Nextera Mate Pair Sample Prep Kit; the mate-pair libraries were bar-coded. The libraries were constructed as per the manufacturer’s instructions. Sequencing was performed by the University of Colorado Denver Genomics and Microarray Core on an Illumina HiSeq 2000 Sequencing System, as per the manufacturer’s instructions. Sequencing utilized four flow-cell lanes per strain for the paired-end library and two flow-cell lanes for all four of the mate-pair libraries. The total number of reads for each library can be seen in Supplemental Table S-1.

The raw reads from the paired-end short insert library were mapped to the reference genome GRCm38/mm10 (mm10) using the BWA aligner (v. 0.5.9) (Li and Durbin 2009). A paired-end mapping strategy with default parameters was utilized, setting the maximum insert size to 1000 (expected insert size 300). After mapping, the reads were sorted and converted into binary alignment format (BAM) via Samtools (v. 0.1.18; Li et al. 2009). The sorted binary alignments then underwent post-processing to remove duplicates via Picard’s MarkDuplicates (v. 1.72; http://broadinstitute.github.io/picard) and local realignment around indels using the Genome Analysis Toolkit (GATK; v. 2.4-9; McKenna et al. 2010).

For the large insert mate-pair libraries, the adapter was clipped from raw reads using FastX (v0.0.13.2; hannonlab.cshl.edu/fastx_toolkit/) and reads shorter than 16 base pairs were removed. The remaining reads were reverse complemented to obtain the forward–reverse orientation required for most downstream analysis programs. Reads were then mapped to mm10 via BWA (v0.5.9) using appropriate insert size settings (10 kb library max size—20,000; 4 kb library max size—8000).

### ILS/ISS variant analysis

The short insert DNA seq libraries were used to call single-nucleotide polymorphisms (SNPs) and small insertions and deletions (indels) less than 50 base pairs with respect to mm10 using the GATK Unified Genotyper with dbSNP build 137. The initial SNP and indel call set consisted of 9,237,224 combined variants from the ILS and ISS. To minimize false positives, this set was filtered using the GATK Variant Quality Score Recalibrator (VQSR) and apparent heterozygous positions were removed based on the GATK Allelic Depth filter. Initially, a coverage depth of five reads and a minimum 90 % of reads over any called variant locus were required to support the variant allele.

Variants were classified in each strain as either common (same variant in both strains, but different from mm10) or strain-distinct (different between the ILS and ISS). For common SNPs, a minimum of five reads were required in each strain with a minimum of 90 % of the reads in support of the variant. Candidate strain-distinct variants were cross-checked with the unfiltered raw variant set from the other strain (the strain without the candidate strain-distinct variant). If the read coverage in the other strain had a minimum of three reads and at least 50 % of these reads had the same sequence as the candidate strain-distinct variant, we no longer consider this variant as strain-distinct or common. Variants overlapping or within plus or minus five base pairs of a repeat region of mm10 (lowercase in the UCSC mm10 fasta file) were removed. The high-quality variants were then compared to ENSEMBL reference annotations to identify those that occurred within genes. Individual ILS and ISS genome and gene annotation files were generated using Seqnature (v 1.0; Munger et al. 2014) incorporating the most high-confidence set of SNPs and small indels for each strain into the mm10 reference (common and strain-distinct).

Large structural variations (SVs; >50 bp) were called using the SVMerge pipeline (v1.2r37; Wong et al. 2010) which integrates results from multiple SV callers: BreakDancerMax (v1.1.2; Chen et al. 2009) was run independently on each DNA sequencing library (10 kb, 4 kb, 300 bp) to detect insertions, deletions, inversions, and translocations; Pindel (v0.2.3; Ye et al. 2009) was run utilizing all three libraries (10 kb, 4 kb, 300 bp) in a single run to detect insertions, deletions, tandem duplications, and inversions; SECluster, a component of the SVMerge package (v1.2r37; Wong et al. 2010), utilizes paired-end reads where only one read in the pair maps to detect potential large insertions; and the short insert paired-end library was used with CNVnator (v0.3; Abyzov et al. 2011) to detect potential copy number gain and losses. After all SV detection programs were run independently, the SV calls were then filtered and merged across redundant calls (i.e., overlapping) using the SVMerge pipeline (v1.2r37; Wong et al. 2010) to produce a final set of SVs which were then subjected to de novo assembly using Velvet (v1.2.07; Zerbino and Birney 2008). Assembled contigs were aligned back to the reference genome using exonerate (v2.2.0; Slater and Birney 2005). SV calls overlapping telomeric regions were excluded from further consideration. Strain-specific SV events were determined as described in Supplemental Methods.

### Ancestor inference

A hidden Markov model (HMM) approach was used to infer the likely ancestral origin of each segment of the ILS and ISS genomes using sequence data from 6 of the 8 original ancestor strains: A, AK, BALB/c, C3H, C57BL, and DBA/2 (Keane et al. 2011); the Is/Bi and RIII have not been sequenced and DNA from the Is/Bi is not available. Our HMM consisted of six states: one state for each sequenced ancestor and one (Unk/C57) that captures both the “unknown” (unsequenced) ancestors and C57BL. Because C57BL is assumed to be genotypically nearly identical to the mm10 reference genome (C57BL/6 J), it was underrepresented in the SNP sets and therefore lacked sufficient support to be a distinct state.

The fully probabilistic treatment of the HMM allows the model to capture key features of the strain derivation. Conceptually, emissions capture not only distinct ancestor biases but also sequencing error and de novo mutations. Transitions between the states correspond to recombination events in the breeding history of ILS and ISS strains. Therefore, fine-scale mouse recombination rates (Brunschwig et al. 2012) were incorporated as positional priors to these transitions. Optimal emission and transition rates were found using an Expectation-Maximization (EM) algorithm (Dempster et al. 1977) with the previously sequenced ancestor strain SNPs and the ILS or ISS strain-distinct SNPs as input. At each EM iteration, regions with identical SNP coverage across multiple ancestor strains are re-labeled as identical by descent (IBD) for those ancestors. Upon convergence, the final maximum-likelihood path yielded the haploblock ancestral origins of the highest confidence. The model accuracy was assessed by consistency of indel variations between the ILS/ISS strain and the inferred ancestral strain. A manuscript describing the model is currently being prepared.

### Quantitative RNA sequencing and analysis

Mice were administered normal saline (0.01 ml/g) and sacrificed 8 h later by CO_2_ inhalation followed by decapitation. The brain was removed and further dissected into cerebellum and whole brain (minus the olfactory bulbs), and stored in RNALater at −20°C until RNA extraction. The RNA-seq studies reported here only used the whole brain sample. Total RNA was extracted using RNeasy Mini Kit (Qiagen, Valencia, CA), and quantity and quality were determined using a NanoDrop™ spectrophotometer (Thermo Fisher Scientific, Wilmington, DE) and Agilent 2100 BioAnalyzer™ (Agilent Technologies, Santa Clara, CA). Ratios of absorbance at 260 and 280 nm were shown to be excellent (>1.8), and RNA Integrity scores were also shown to be excellent (>8.0). Total RNA was stored at −80°C until library preparation.

Total RNA was isolated from nine mice per strain and an equal amount of RNA from three mice of the same strain was pooled for each library; thus, three libraries per strain were prepared. Pooling in this manner reduces within-strain variance which produces an effective increase in statistical power without increasing the number of libraries (Kendziorski et al. 2005; Kendziorski and Wang 2006). Samples were enriched for poly-A RNA using the Dynabeads mRNA Purification kit (Invitrogen) as directed by the manufacturer. Paired-end (2 × 100, expected size of 300 bp), strand-specific, cluster-ready libraries were prepared from the poly-A-enriched RNA using the ScriptSeq RNA-Seq Library Preparation Kit v2 (Illumina), following the manufacturer’s instructions. Sequencing was performed by the University of Colorado Denver Genomics and Microarray Core on an Illumina HiSeq 2000 Sequencing System as per the manufacturer’s instructions with 6 bar-coded libraries pooled per flow-cell lane. The total number of reads for each library can be seen in Supplementary Table S-2. We note that the ISS libraries produced approximately twice as many reads as the ILS. We believe that this was unlikely to be a biological effect and the various procedures were scheduled in such a way as to essentially eliminate any kind of strain-specific batch effects, i.e., each of the dissections and RNA isolations were conducted on completely different days, while the library preps and sequencing were conducted on four different occasions with an ILS and an ISS sample paired on two of the occasions and the remaining two ILS and ISS libraries were prepped and sequenced on completely different days with other samples not related to this study. All of the dissections, RNA isolations, and library preps were performed using the same protocols and reagents, and by the same person. In addition, there did not appear to be any difference with regard to mapping parameters (see Results). We can therefore only conclude that the difference in total reads was a random effect.

RNA sequencing data were mapped back to both the reference mm10 genome and the strain-specific genomes (ILS and ISS) using TopHat2 (v2.06; Kim et al. 2013) using their respective transcriptome annotation files. TopHat2 was run using the b2-very-sensitive option, allowing for microexons but not novel junctions. A custom script was used to compare read mapping locations in mm10 versus the strain-specific genome.

Whole-gene quantification was determined using HTSeq (v0.6.1; Anders et al. 2015) which provides raw read counts over an annotated gene set. Only uniquely mapping reads were used for quantification. Raw read counts were then used as DESeq input for differential gene expression analysis (v1.10.1; Anders and Huber 2010). The standard DESeq workflow was followed. Genes having an adjusted *p* value (Benjamini and Hochberg 1995) of 0.1 or less were considered significantly differentially expressed.

In order to quantify the impact of mapping to the strain-specific genomes versus to the reference genome (mm10), custom scripts were used to compare the whole-gene count files (from HTSeq) on a gene-by-gene basis to investigate the change in read counts when mapping between genome versions. As coordinates may shift in strain-specific genomes, comparisons were based on DESeq identifiers for whole genes and DEXSeq identifiers for exonic regions. Exon quantification was performed using DEXSeq (v1.4.0; Anders et al. 2012) using the standard workflow.

## Results

We obtained over one billion raw reads for each of the ILS and ISS genomes, and approximately 81 % of the reads remained in each of the strains after filtering for low-quality and duplicate reads (Supplemental Tables S-1 and S-3). There was no significant difference in mapping quality between the different libraries, as suggested by the finding that approximately 95 % of the filtered reads mapped to mm10 (Supplemental Table S-3). This resulted in sequencing of the ILS and ISS genomes to 28.6-fold and 30.7-fold coverage, respectively. Consistent with the ILS and ISS strains being highly inbred, the majority of sequenced SNPs and small indels were homozygous (99.89 % in ISS and 99.86 % in ILS). The small amount of heterozygosity likely primarily arises from collapsing reads from duplicated regions within the genome, as heterozygous variants tend to cluster in repetitive regions of the genome. It is also possible that a small number arise from incomplete fixation during breeding or recent de novo mutations.

Over 4 × 10^6^ high-quality common and strain-specific SNPs and small indels (<50 bp) were identified using just the short insert library (Table 1; see link to file for the complete list; note that a small number of these variants have been published previously in Bennett et al. 2015). We assessed the quality of the SNPs by comparison to a set of 43,870 informative ILS/ISS SNPs generated by Churchill and colleagues at The Jackson Laboratory using the Affymetrix Mouse Diversity Genotyping Array (see Saba et al. 2011). In total, 99.7 % of the microarray-identified SNPs were observed within our set of unfiltered SNPs; the small number that did not confirm were due to a variety of issues (see Supplemental Table S-4). When comparing the array markers to our final filtered SNPs, we found an overlap of 96.8 % suggesting that our final filtered variant set is quite stringent.

**Table 1:** Summary of genome-wide variants identified in the ILS and ISS using the short insert paired-end library

Variant type^a^	ILS^b^	ISS^c^	Common^d^
Total	1,582,616	1,114,887	1,434,163
SNPs	1,346,137	943,224	1,226,435
Deletions	118,831	87,076	107,161
Insertions	117,648	84,587	100,567
Coding total	15,472	10,732	15,219
Synonymous	9833	7140	9563
Non-synonymous	5391	3421	5451
Coding deletions	57	39	53
Coding insertions	47	39	44
Coding frameshift	108	74	87
Coding stop	36	19	21
dbSNP	1,372,946	949,884	1,281,181
Private (not in dbSNP)	209,670	165,003	152,982

^a^The variant type is in comparison to the reference

^b^Different than both ISS and reference

^c^Different than both ILS and reference

^d^Same in ILS and ISS, different than reference

We also compared, by manual inspection, our unfiltered variants to a set of 7438 strain-specific variants identified by Dumas et al. (2014) through exome sequencing. Most of these SNPs were also within our unfiltered list (98 % in ILS and 99 % in ISS). The missed SNPs either overlapped repeat regions or had no evidence in our whole-genome sequencing. Frameshifting indels were confirmed at a lower efficiency (58 % ILS; 76 % ISS), likely reflecting the relative difficulty in detecting these events with either technology. Interestingly, many of the unconfirmed exome sequencing variants appear heterozygous in our whole-genome sequencing data. These heterozygous variants could be indicative of potential CNVs. Indeed, of the 60 heterozygous positions, 12 overlap with a called CNV from the SVMerge pipeline.

**Fig. 1:**
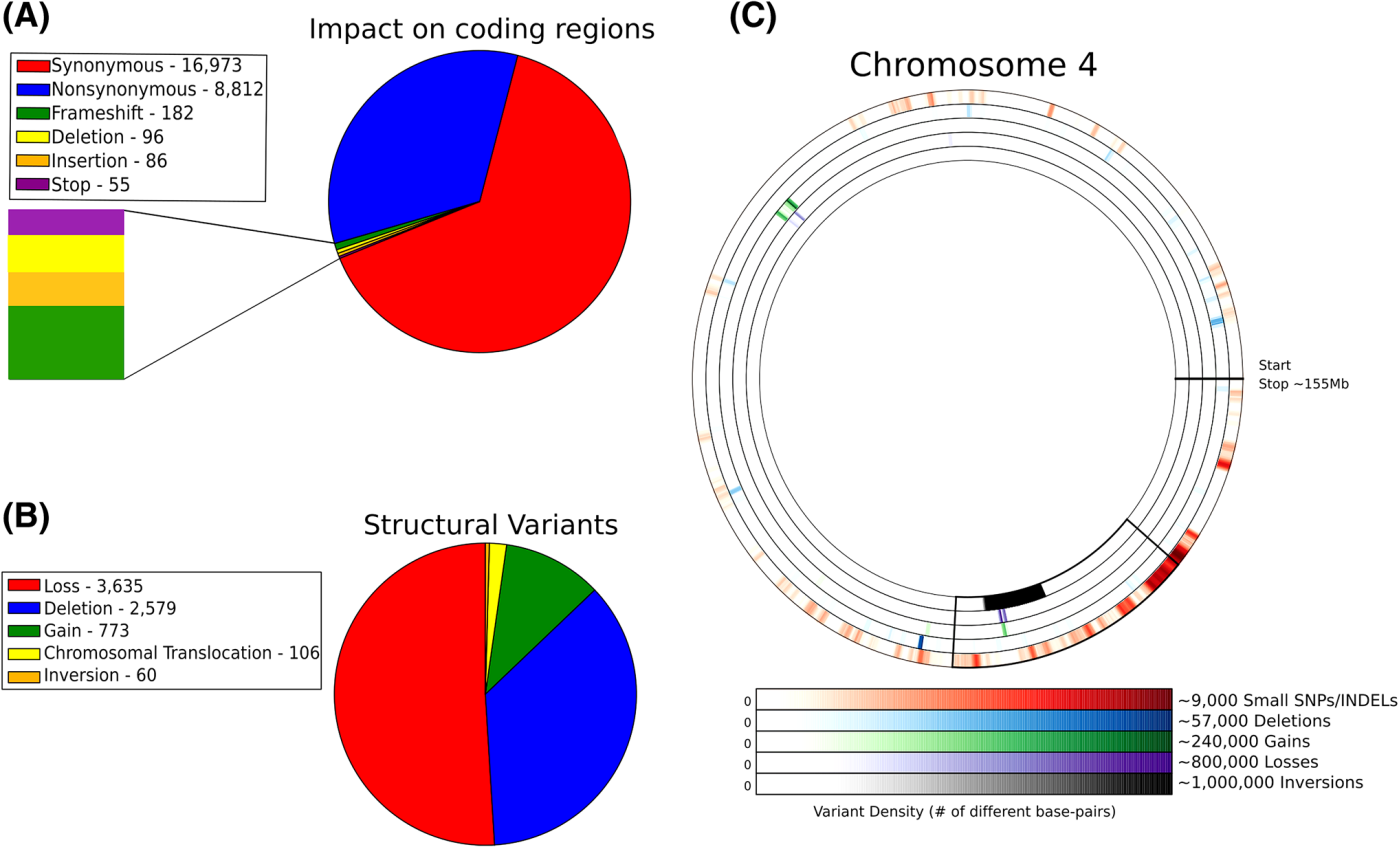
Differences between ILS and ISS strains: Variant Breakdown. **a** Combined totals of variants that differ between the ILS and ISS strains. Synonymous mutations refer to a SNP(s) that alters the codon sequence but not the amino acid produced. Synonymous and non-synonymous mutations arise from the impact of a SNP on the underlying codon. Frameshifts are any indel not divisible by three, whereas deletions and insertions retain the reading frame. Variants are counted once per gene. **b** Combined totals of structural variations that differ between ILS and ISS strains. Losses and gains refer to changes in copy number, whereas a deletion is a complete loss/absence of a region. Chromosomal translocations refer to exchanges of large segments between chromosomes and inversions are reversals. **c** Distribution of structural variations by type, summarized by a sliding window approach (500 k windows with 100 k step size) (Color figure online)

Our filtered, highest confidence set of variants included a total of 2,697,503 strain-distinct SNPs and small indels, which are distributed across the genome (Table 1; see Supplemental Figure S-2). Approximately 87 and 85 % of the strain-specific SNPs and indels were found in dbSNP (build 37) in the ILS and ISS, respectively, suggesting that the remainder have not previously been detected in any other sequenced mouse strain. We observe that the strain-specific variants (SNPs only) have a Ti/Tv ratio of 1.5 and 1.7 for ILS and ISS, respectively. These strain-distinct variations impact 5911 annotated genes, including 236 that result in disruption of an open reading frame through frameshifts or the introduction of a stop codon (Table 1; Fig. 1a).

Similar to our SNP analysis, we sought to identify the highest confidence set of strain-distinguishing SVs, i.e., those that differ between the ILS and ISS. To this end, we developed a novel method for scoring and selecting strain-distinct events. SVs identified in either strain were scored for reads supporting the event in both strain backgrounds. We then identified the score cutoff that optimizes the false discovery rate of strain-unique events (Supplemental Figure S-3 and Table S-5; see Supplemental Methods for complete details), resulting in 7153 strain-specific events (Table 2; Fig. 1b, c; Supplemental Figure S-2). By manual inspection, we found good correspondence to copy number variants (CNVs) that were previously identified by arrayCGH (Dumas et al. 2014). A third of the previously called CNVs were confirmed (same call within ± 600 bp of breakpoints) using our pipeline. In another third of cases, our pipeline identified some structural variation within the region (±600 bp of breakpoints) though the label of the aberration differed. For the remaining third, we found no evidence of the CNVs in the sequencing data.

**Table 2:** Summary of genome-wide structural variants identified in the ILS and ISS

Variant type^a^	ILS^b^	ISS^c^
Total	4271	2882
Deletions	1488	1091
Gains	527	246
Losses	2168	1467
Inversions	29	31
Translocations	59	47

^a^The type of variant, in comparison to the reference

^b^Different than both ISS and reference

^c^Different than both ILS and reference

**Fig. 2:**
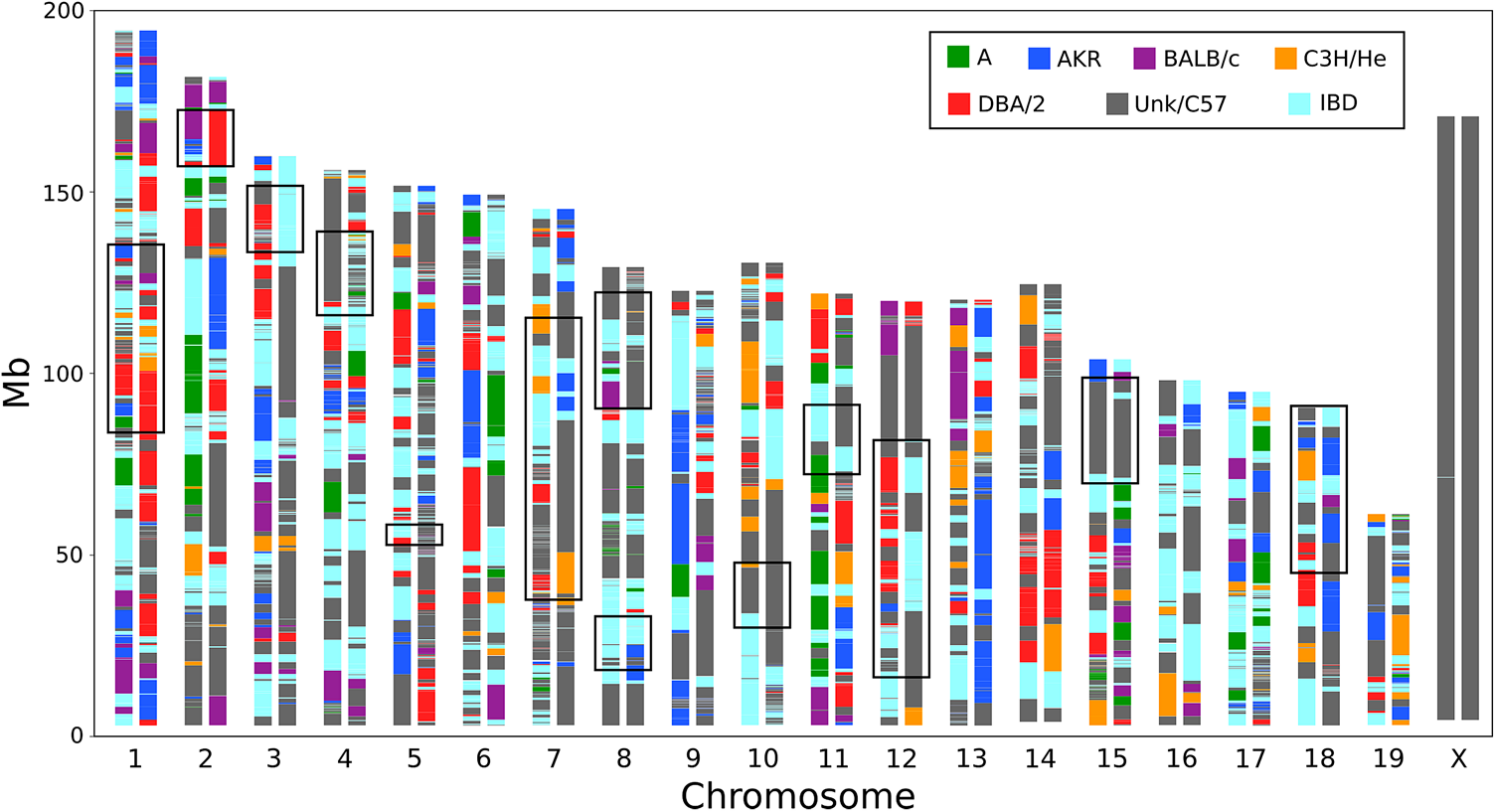
Chromosomal breakdown of ancestor inference. For each chromosome, distributions of inferred ancestry in the ILS (on *left*) and ISS (on *right*) genomes. QTL regions (as listed in Table S6) are *boxed* (Color figure online)

The inferred ancestry for the whole genome as determined by our HMM is shown in Fig. 2. On a per-chromosome basis, we observed distinct patterns in the inferred ancestor origin. In ILS and ISS, the three strains represented by Unk/C57 make up approximately 2/5 and 1/2 of the total distribution, respectively (Supplemental Figure S-4). Aside from Unk/C57, we found that each other ancestor contributed approximately evenly to both ISS and ILS genome wide. We note that chromosome X for both strains is classified almost completely as Unk/C57. Approximately 23 % of the SNPs found in each of the X chromosomes could, in fact, be assigned to one of the ancestral strains, but these SNPs were distributed in a way that prevented the HMM from detecting ancestral blocks of any significant size. Also, it is possible that the X chromosome was fixed either during a severe reduction in fertility and thus in breeding families that occurred early on during the selection of the LS and SS (McClearn and Kakihana 1981) or during the inbreeding of the ILS and ISS. We have full confidence that our sequencing analysis and the HMM were correct; however, the fact that such a high proportion of chromosome X was Unk/C57 for both strains is difficult to explain.

**Fig. 3:**
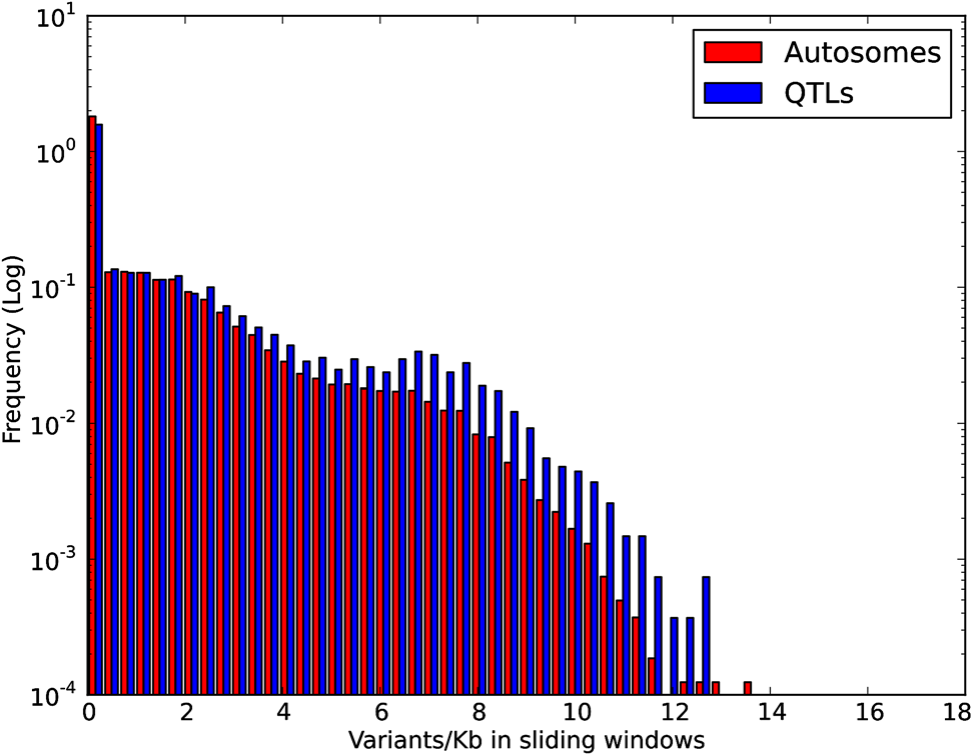
QTLs have an elevated variant density. Histogram summarizing the variant density (variants/kb) throughout the genome on autosomes. *Red* for non-QTL regions; *blue* for QTL regions (Color figure online)

Quantitative trait loci (QTLs) have been mapped for a variety of traits in the LXS and other segregating populations derived from the ILS and ISS. Here we restrict our analysis to those QTLs mapped for sleep time, the trait on which the ILS and ISS were selected, and other closely related traits such as acute and rapid tolerance for sleep time (Supplemental Table S-6). We found that QTLs were more variant dense than the rest of the genome despite several QTLs that were mostly identical by descent (IBD) (Fig. 3; Supplemental Figures S-2 and S-5). As one might expect, within any given QTL, there is a difference between the ILS and ISS in the ancestral makeup of the QTL region (Fig. 2).

In order to determine the effect of mapping to a particular genome sequence on RNA-seq quantification, we conducted a differential expression (DE) analysis on RNA-seq data that were aligned to the mouse reference genome from the C57BL/6 strain (mm10), and to the strain-specific ILS and ISS genomes. Total reads mapped were slightly lower when mapped to the reference genome compared to the strain-specific genomes (71 vs. 72 % and 69 vs. 70 % in the ILS and ISS, respectively); the same was true of uniquely mapped reads (Supplemental Table S-7). The majority of uniquely mapping reads did not change in mapping status or position; however, there was a net gain in unique reads of 1.6 and 1.5 % in the ILS and ISS, respectively. Additionally, a small number of unique reads changed in location when mapped to the strain-specific genome, but remained unique (<0.1 %; Supplemental Table S-7).

**Fig. 4:**
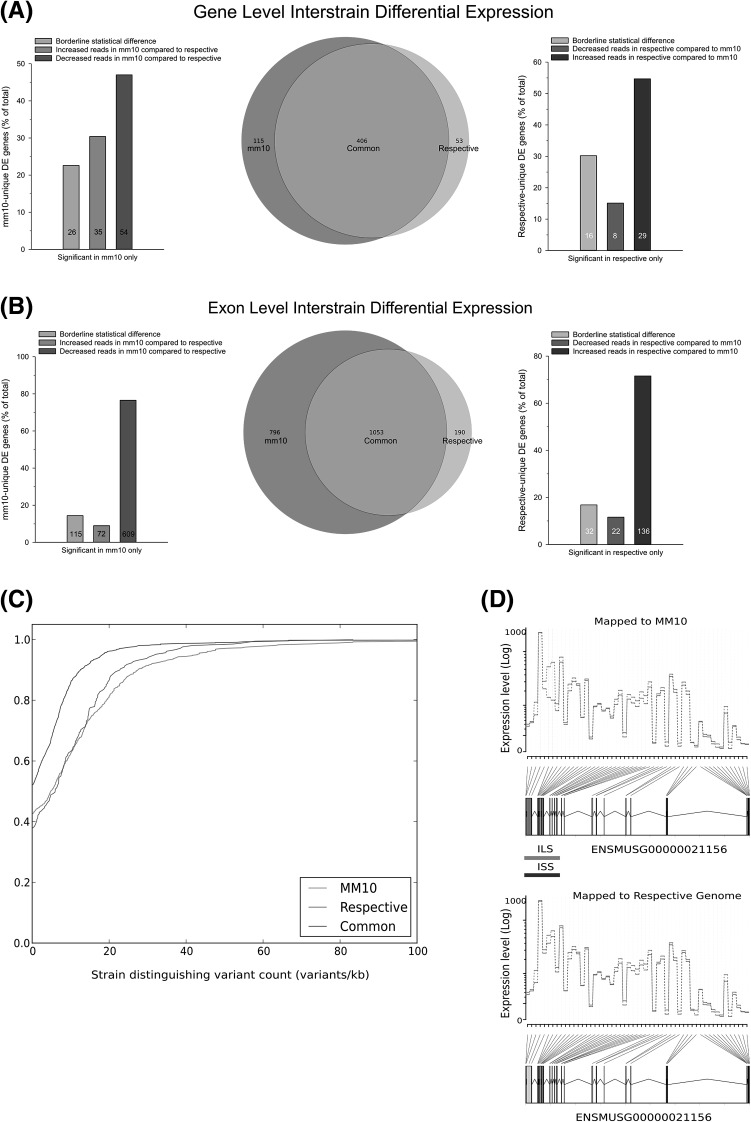
Impact of distinct reference genomes on differential expression. **a** Venn diagram comparing differential expression results between ILS and ISS whole brain RNA-seq samples when mapped to the reference genome (mm10,* red*) versus strain-specific genomes created from SeqNature (*green*). *Bar charts* show fraction of genes that were borderline to cutoff, increased or decreased when showing mapping-specific significance. **b** Venn diagram comparing DEXSeq-annotated exonic binned differential expression results when mapped to mm10 versus the strain-specific genomes. **c** CDF plot of strain-distinguishing variants over DEXSeq-annotated exonic bins for genes considered differentially expressed only when mapped to mm10 (*red*), only when mapped to strain-specific genomes (*green*), and common to both mappings (*blue*). **d** DEXSeq gene plot showing expression level (*y-axis*) for each exon (*x-axis*) for ENSMUSG00000021156 mapped to mm10 (above) and strain-specific genomes (below). Exonic bins considered differentially expressed against mm10 are no longer considered differentially expressed when mapped to the strain-specific genomes (Color figure online)

In a comparison of ILS to ISS gene expression, 521 and 459 genes that had been mapped to mm10 or to the ILS/ISS genomes, respectively, were found to be differentially expressed (DE; Fig. 4a). The majority of DE genes (406) were unaffected by the genome used for mapping; however, under the statistical parameters used, 115 DE genes were lost and 53 were gained in the strain-specific genome analysis relative to the mm10 analysis. The majority of changes arose from more reads mapping to the strain-specific genomes than mm10, as illustrated in the bar graphs in Fig. 4a; however, many of the genes were lost because they shifted from just barely significant to just barely non-significant based on the statistical cutoff, or vice versa (23 % of the significant mm10 DE genes and 30 % of the significant strain-specific DE genes). In addition, a portion of the genes had higher read counts when mapped to mm10 than to the strain-specific genomes; nearly all those that were significant were pseudogenes (34/35), whereas only 3 of the 8 strain-specific genome DE genes were pseudogenes. Given that the strain-specific genomes more accurately reflect the underlying genotype of the strains, we note that 115 genes were false positives and 53 genes were false negative when differential expression was called relative to mm10 (Fig. 4a).

We next conducted a similar analysis on exon usage. Overall, the pattern compared to the full gene analysis was similar, although the percentage of mm10 DE exons as a proportion of total mm10 DE exons was substantially higher than for DE genes (43 vs. 22 %; Fig. 4b). In addition, a much higher percentage of DE exons were found to increase in reads when mapped to the strain-specific genome versus mm10 compared to the full gene analysis (bar graphs in Fig. 4b). Perhaps unsurprisingly, the exons whose DE is genome dependent were enriched for variants compared to common DE exons (Fig. 4c). These SNP-dense regions often gain additional reads when mapped to the strain-specific genomes, which can result in a loss of DE exons (Fig. 4d), but also in a gain (Supplemental Figure S-6). We also observed that some exons change in DE status despite being absent in strain-specific SNPs. Manual inspection indicated that these exons were adjacent to a SNP-dense exon, which resulted in changes in splice-junction read mapping.

In earlier work, we examined the difference in gene expression between naïve ILS and ISS mice using shorter read lengths (28 bp single-end reads) and a different sequencing strategy (Darlington et al. 2013). We used the current pipeline to determine if there would be a similar mapping genome effect. Similar shifts were observed in the set of DE genes when mapping to the strain-specific genomes rather than to mm10, although the magnitude of impact on exon DE was dampened in the earlier dataset compared to the current study (Supplemental Figure S-7). This is probably a reflection of both shorter read lengths and lower depth of coverage in the earlier study. We also compared DE genes in the current study to those identified in the earlier study after running both datasets through an identical pipeline using the strain-specific genomes. Approximately half of the DE genes from each analysis were found to be in common (Supplemental Figure S-7).

## Discussion

Up until fairly recently, the field of quantitative genetics—now generally referred to as *complex traits analysis*—was mostly descriptive at the phenotypic level. As a result of the development of high-throughput technologies such as expression microarrays and NextGen deep sequencing, and also novel computational approaches, complex traits analysis has moved increasingly toward the molecular level. Here, we have used NextGen deep sequencing to characterize the genomes of a well-established mouse model of acute alcohol sensitivity, the ILS and ISS. The two main objectives of this study were to provide insight into the genomic variants that could contribute to the enormous phenotypic difference between the ILS and ISS, and to generate ILS/ISS reference genomes for accurate alignment and quantification of RNA-seq data from an ongoing acute alcohol experiment with the ILS, ISS, and the LXS RI panel.

Our complete genome sequence refines our understanding of the distinct differences between the ILS and ISS strains. We identified a large number of strain-specific sequence variants that can be used as markers to distinguish between the strains and, importantly, to help elucidate the genetic factors that contribute to the difference in acute alcohol sensitivity that exists between these strains. Previous work had identified ~40,000 SNPs (Saba et al. 2011) and fewer than 100 SVs (Dumas et al. 2014); here we report ~2.7 million SNPs and small indels, and over 7000 SVs that are different between the ILS and ISS, greatly expanding our knowledge of potential variants that contribute to phenotypic differences between the strains and among the LXS RIs. In addition, we have identified ~375,000 SNPs that are not present in dbSNP, implying that they have not previously been detected in any other sequenced mouse strains. Given that six of the eight progenitors of the ILS and ISS strains have been sequenced, this set of private variants likely contains both de novo mutations and variants specific to the missing sequence of the two ancestors, Is/Bi and RIII.

Our unfiltered set of SNPs and small indels is comparable to the number of variants observed by Keane et al. (2011) who used a less stringent filtering procedure; filtering reduced that number by more than half. Moreover, almost 100 % of our unfiltered SNPs were consistent from the ILS/ISS Mouse Diversity Genotyping Array results and about 3 % of those SNPs were lost after filtering. Therefore, our final set of high-confidence variants likely underestimates the actual number. We chose to go in a more conservative direction in order to generate high-quality genomes for mapping RNA-seq reads to the LXS RI strains, of which the ILS and ISS are the progenitors; this is discussed in more detail below.

Our sequencing of the ILS and ISS revealed a large number of DNA variants, of which some number mediate the phenotypic difference in sleep time between the strains. One principal way in which this could occur is through an effect on the structure of gene products. Conservatively, we identified over 9000 SNPs and small indels that affect protein structure and an additional nearly 17,000 non-synonymous SNPs that may affect protein folding or trafficking. We also identified over 6000 large structural variants that potentially affect protein structure, although confidence with those events is lower due to the difficulty in their ascertainment.

A second mechanism through which DNA variants could affect phenotypic outcome is through modulation of the abundance of proteins. For example, SNPs found in promoters or regulatory elements can influence the expression of mRNA and there are numerous other mechanisms through which transcription or translation can be regulated. Although much less is known about how DNA variants affect the regulation of expression than protein structure, our sequencing results can be used in combination with our ongoing LXS RNA-seq experiment to gain insight into the variants that mediate differences in expression.

The ILS and ISS were generated from a heterogeneous population derived from 8 inbred mouse strains, 6 of which are widely used and have been sequenced (Keane et al. 2011). These 6 strains—C57BL, A, AK, BALB/c, C3H, and DBA/2—are closely related, especially the latter 5 which all were derived from a single line of Castle’s mice (Beck et al. 2000). This is consistent with our ancestor inference analysis, which indicates that approximately 25 % of the ILS and ISS genomes are IBD with respect to these 5 strains. In contrast, 50 % of the ISS genome comprises only 3 of the strains—the C57BL and the unsequenced Is/Bi and RIII (Unk/C57)—which could not be further distinguished, as described in Methods section. This is higher than the 37.5 % that would be expected by chance if all 8 strains were more or less equally represented. Interestingly, in 9 of the 15 QTLs related to sleep time, substantially more of the genome comes from Unk/C57 group than the other 5 strains combined. This is due more to the genetic variation contributed by the Is/BI and/or RIII than from the C57BL as mapping studies using crosses derived from the C57BL/6 and DBA/2 strains show limited genetic variation compared to the LXS RIs (Radcliffe et al. 2000). Perhaps, this is expected since the Is/BI and RIII are very unrelated to the other 6 strains, including, apparently, the founder breeding pair for the Is/BI that includes a wild mouse captured on an Israeli dock (Beck et al. 2000), i.e., these two strains contributed as much or more genetic variance to the ILS and ISS than all of the other 6 strains. Should the RIII strain, which is still readily available, be sequenced it could be used to both further validate our ancestor inference and identify causal variants within QTL regions.

Typically, only a fraction of the genetic variance is accounted for in a QTL experiment. Part of this is probably a result of the difficulty to detect epistatic interactions, which generally requires a large mapping population. Another possibility is that some portion of the genetic variance is mediated by a large number of small-effect genes, i.e., the infinitesimal model (Fisher 1918). If much of the genetic variance between the ILS and ISS fits this model, it would explain why we observed an enrichment of variants in QTL regions. If trait-relevant variants were equally distributed among all variants, only variant-dense regions would contain enough genes for their additive effect to be detected as a QTL. Moreover, it appears that there is not a particularly greater rate of recombination in QTL regions than elsewhere in the genome based on our ancestor inference analysis. Indeed, the genome was derived almost entirely from only one or two strains for some of the QTLs (e.g., Lore5, QTL-A, and QTL-B for ILS, and Lore3, Lore5, QTL-C, and QTL-E for ISS) and nearly all of the QTLs show ancestral distributions that are both inconsistent with equal strain distribution and unlike the strain distribution genome wide. This is consistent with a model in which relatively large haplotype blocks from each of the ancestors contained many linked small-effect genes that move the phenotype in the same direction and therefore remained intact during the selection process (Barton and Keightley 2002). The remainder of the genetic variance not accounted for by the mapped QTLs would then be more or less randomly distributed throughout the genome.

Early “genetical genomics” experiments that employed hybridization microarrays to quantify whole-genome gene expression tended to find an unusually high number of *cis*-regulated expression QTLs (eQTLS; see Peirce et al. 2006). It was realized that many of these *cis*-eQTLs were resulting from “SNP hybridization artifacts,” i.e., expression levels were artificially reduced by poor hybridization of SNP-containing mRNA fragments from genotypes that differed from the reference genome, the C57BL/6, and, of course, the expression level segregated with the SNP in the mapping population leading to a false-positive *cis*-eQTL (Walter et al. 2007). A similar phenomenon occurs with RNA-seq, although it is a computational rather than physio-chemical effect as with microarrays. Munger et al. (2014) examined the impact of mapping to the reference on gene quantification and eQTLs for mouse strains, finding that the identity of the reference had a dramatic impact on a small number of loci (but see Panousis et al. 2014). Here we have extended this effort to examine the impact of the reference on differential expression. Despite the fact that mapping to the strain-specific genomes influenced only a small number of reads, we found that this effect impacted a relatively large number of genes and exons. Consistent with Munger et al. (2014), we found that pseudogenes are notorious for creating this type of artifact, and much of it becomes resolved with mapping to the correct genome. This is also partly due to the particular statistical cutoff that is used, i.e., approximately 25 to 30 % of the DE genes affected by mapping genome were just barely significant or non-significant. The percentage was less with exons because of their much smaller size, which makes them more sensitive to the effect. With a more or less stringent statistical cutoff, these genes would be nearly completely excluded or included, respectively; however, for approximately 20 % of all DE genes, the effect is robust and independent of statistical issues. This is a higher value than that determined by Bottomly et al. (2011) in a similar analysis of the C57BL/6 and DBA/2 mouse strains. The difference can be explained by two technical distinctions: Bottomly et al. (2011) did not include small indels in their analysis and they used a considerably shorter read length (43 vs. 100 bp). Regarding the latter issue, shorter read lengths are less likely to overlap a SNP but are also less likely to be unique (mappability). Additionally, the magnitude of difference likely depends on the distance (extent of variation) between the strain and the reference.

We have identified a large number of variants between the ILS and ISS that were bidirectionally selected for acute alcohol sensitivity. Even though these lines underwent an artificial selection procedure, the genetic variation between them is generally similar to that between any two randomly chosen standard laboratory mouse strains (Keane et al. 2011). It is certainly true that only a subset of the DNA variants contributes to the enormous difference in alcohol sensitivity, i.e., many of the variants segregated randomly or as a result of linkage and mediate genetic variation for the large number of phenotypic differences between these lines. Therefore, the catalog of DNA variants alone does not definitively provide us with the relevant alcohol sensitivity genes, it does provide us with candidates that can be further refined using techniques such as QTL mapping and other systems genetics analyses. Moreover, we now have the ability to more effectively map RNA-seq reads for our large ongoing LXS study which will also help identify, or at least narrow down, the genes that are contributing to acute alcohol sensitivity. It may be, however, that the phenotypic difference is the result of many small-effect genes which we have previously argued is the case for an important QTL that maps to distal chromosome 4 (Bennett et al. 2015). One primary goal of QTL mapping is to identify the “QT gene”; however, this may be possible for only a limited number of genes that have a large enough effect. Nonetheless, there may be some hope for the successful dissection of the ILS/ISS QTLs by conducting careful functional enrichment analyses with the RNA-seq data that we generate from the LXS RI panel: the effect of any individual gene among many may not be tractable, but the effect of a handful of functionally related gene clusters may be. The current work contributes substantially to that endeavor.

## Electronic supplementary material

Below is the link to the electronic supplementary material.

Supplementary material 1 (PDF 5994 kb)
