# Supplementary material for: Genome characterization of the selected long- and short-sleep mouse lines
Source: Mamm Genome. 2016 Sep 20;27(11):574–86. doi: 10.1007/s00335-016-9663-6 (PMC5110614; doi:10.1007/s00335-016-9663-6)
Supplement: Supplementary file 1 — Supplementary material 1 (PDF 5994 kb) [file 335_2016_9663_MOESM1_ESM.pdf]

SUPPLEMENTAL METHODS

Mapping differences

In order to compare RNA seq read mapping locations between the reference mm10 genome and the strain-specific genomes a custom script was written. The script identifies the mapping location and status (unique vs non-unique mapping) of each read in the mm10 mapped RNA seq file and compares these to the strain-specific mapped RNA seq file reads. If the relative mapping locations and or status change between mapping to mm10 vs mapping to strain-specific then this change is noted (Supplemental Table 7). Because the libraries are paired-end, comparisons were performed on the first and second read of each pair independently as denoted by the R1 and R2 nomenclature.

Detailed HMM methods

We sought to infer the ancestral origin of distinct regions within both the ILS and ISS genomes. The ILS and ISS strains were selectively bred for their response to alcohol from eight ancestor strains: A, AK, BALB/c, C3H, C57BL, DBA/2, Is/Bi, and RIII [McClearn, 1981]. Of the ancestor strains, A, AK, BALB/c, C3H, C57BL, and DBA/2 have been previously sequenced and genotyped [Keane, 2011], while Is/Bi and RIII have not. As the sequenced ancestor strains lack information for the Y chromosome, we removed it from our analysis of ILS and ISS. The strain designations used here are based on Keane et. al. [Keane, 2011].

Intuitively, we can say that regions of the ISS and ILS genome with SNPs largely consistent with variants from a single ancestor were likely inherited from that ancestor. Using a hidden Markov model (HMM) approach we can probabilistically infer the most likely ancestral origin of each genome segment. Because C57BL is genotypically very similar to the mm10 reference genome, it was underrepresented in the SNP sets and very sensitive to *de novo* mutations in the dataset. Because of this, we combined C57BL and the Unknown into a single state, Unk/C57. Therefore our HMM consists of six states: one state for each sequenced ancestor and one "Unknown/C57" state to capture the two unsequenced ancestors and C57BL.

For our HMM training set, we use all ILS or ISS high quality SNP positions and the SNP positions of the six sequenced ancestor strains. Conceptually, transitions between distinct states correspond to recombination events during breeding, while transitions to the same state indicate adjacent SNPs belong to the same ancestral haploblock. Therefore, finding a maximum-likelihood path

through the HMM results in the haploblock ancestral origins of the highest confidence.

The model parameters are set initially using biological intuition and refined using Expectation-Maximization. State emissions report on the consistency of the observed ILS or ISS strain with the designated ancestor. For example, when running the HMM for the ILS strain, if a given position in the genome contains a SNP consistent with the DBA/2 ancestor then state DBA/2 is given a high emission rate for that position. We note that inconsistencies can arise from sequencing errors and *de novo* mutations, thus non-ancestral variations in each state have non-zero probability. As adjacent SNPs are most likely to be from the same inherited region, we set the initial self-transition probabilities high. Additionally, we incorporate fine-scale mouse recombination rates [Brunschwig, 2012] to adjust the transition probabilities in a position specific fashion. As recombination rates are per-base and region-specific, this has the added benefit of naturally incorporating the distance between SNPs into our transition probabilities. To converge on optimal transition and emission probabilities, the HMM is run through an Expectation-Maximization loop. In each iteration, the maximum-likelihood path through the HMM is found using the Viterbi algorithm (maximization step), then transition and emission probabilities are recalculated based on the results (expectation step).

We note that SNPs inherited from one of the unsequenced ancestors will only appear in the data if those SNPs are not consistent with any of the six sequenced ancestor strains. Therefore, if a region was inherited from an unsequenced ancestor, but is genotypically similar to a sequenced ancestor, there is a bias for the HMM to label the region as coming from the sequenced ancestor rather than Unk/C57. We attempt to correct for this by relabeling regions with a certain percentage (we utilized 90%) of unique ISS/ILS SNPs as Unk/C57. Furthermore, we identify regions within the ILS and ISS genomes that are identical by descent (IBD). Sometimes large segments of the ancestor genomes are absolutely identical and indistinguishable. We sought to identify these regions within the HMM output, where a particular ancestor is chosen (by Viterbi) based on little to no informative positions. In these cases, there is no way to truly distinguish the ancestor of origin so we reclassify the segment as IBD for the set of nearly identical ancestors.

To assess our model, each haplotype block classified by the HMM is evaluated for its consistency relative to insertions and deletion (indel) structural variations observed in the ancestor strains. That is, for each haploblock classified by the model, we found all indels in the ILS/ISS strain and the ancestor strains that overlap that haploblock region. Then for each unique indel, we check for

consistency with the model classification and the ancestor indels. We score a "hit" if the indel region contains the indel of the ancestor classified from the HMM and a "miss" if the region does not contain that indel. The final ratio of hits to misses gives the HMM output a score.

Source code for the HMM can be found at the Dowell lab website: http://dowell.colorado.edu/resources.html

SV scoring method

The events output by SVmerge [Wong 2010], namely 22,912 events called in ISS and 25,092 events called in ILS, were further analyzed to determine strain specific structural variants. First, 16,916 candidate strain-specific events were selected from the SVmerge results by identifying those deletions, inversions, translocations, and gains greater than 1 Kb in length called in only ISS or ILS but not both. Events were deemed the same if the start and stop were within 600 bp of each other, otherwise the event was considered strain-specific.

Strain specific SV candidates were scored for paired-end support or coverage support depending upon applicability of the short insert library read data. Paired-end support considers insert size and orientation while coverage support considers read-depth. Paired-end support scores were generated for calls originally identified by discordant paired-end mapping. These included deletions called by BreakDancer [Chen 2009], as well as translocations and inversions. Coverage support scores were generated for events originally identified by read-depth metrics. These included gains and deletions called by CNVnator [Abyzov 2011].

For those events evaluated by paired-end support, the short insert library reads were used as input to the R package targetSeqView [Harper-Stromberg 2014] to generate scores. For those events evaluated by coverage support, coverage was summed within non-overlapping 100 bp windows tiled across the length of each event. The coverage score was the median coverage statistic from all windows across the event.

Score cutoffs for declaring an event to be a high confidence were generated by adapting a method from Ramskold et al., previously used to determine differential gene-expression cutoffs [Ramskold 2009]. This method was originally intended to compare the expression values (in RPKM) of genes versus intergenic 'background' regions. This method has the attraction of attempting to optimize the distinction between two distributions, one with mostly positives (the 'signal'), and one with all negatives ('background'). Our data was similarly constructed as two score distributions. For each candidate event, the score from the mouse of origin was denoted as 'signal' (i.e the mouse where the event was originally called by SVmerge) and a score for the alternate mouse was denoted as 'background' (i.e the same locus from the other strain). The original publication was interested in optimizing the false negative rate whereas the

objective here was to optimize the false discovery rate (FDR). False discovery rate was defined for increments of score value, i:

FDR_i= $cumulative\_alternate\_mouse\_events_i$ $/cumulative\_mouse\_of\_origin\_events_i$ * (1- $cumulative\_mouse\_of\_origin\_events_i$)/(1-$cumulative\_alternate\_mouse\_events_i$).

The latter term was a correction for negatives in the mouse of origin distribution. FDR values were plotted (Fig S-3) against score values and change-points were determined using the changepoint R package with the command: cpt.var(diff(fdrs), method="PELT") [Killick 2013]. Score cutoffs were set to be the change-point in the FDR curve with the greatest absolute difference between the two strains in the number of events exceeding that score cutoff. A strain-specific event was determined to be a high confidence event if the score cutoff was exceeded in one strain but not the other based upon testing the candidate event independently in both mice.

To summarize, a highly confident strain-specific call required strong evidence of strain specificity based upon scores generated from coverage metrics and paired-end support metrics. Based upon the aforementioned threshold criteria, 7,153 strain specific events were categorized as high confidence, namely 6,214 deletions, 773 gains, 60 inversions, and 106 translocations (Table S-5).

References

Abyzov A, Urban AE, Snyder M, Gerstein M: **CNVnator: An approach to discover, genotype, and characterize typical and atypical CNVs from family and population genome sequencing**. *Genome Res* 2011, **21**:974–984.

Bennett B, Johnson TE: Development of congenics for hypnotic sensitivity to ethanol by QTL-marker-assisted counter selection. *Mamm Genome* 1998, 9:969-974.

Bennett B, Beeson M, Gordon L, Carosone-Link P, Johnson TE: Genetic dissection of quantitative trait loci specifying sedative/hypnotic sensitivity to ethanol: mapping with interval-specific congenic recombinant lines. *Alcohol Clin Exp Res* 2002, 26:1615-1624.

Bennett B, Carosone-Link P: Replication of small effect quantitative trait loci for behavioral traits facilitated by estimation of effect size from independent cohorts. *Genes Brain Behav* 2006, 5:404-412.

Bennett B, Carosone-Link P, Zahniser NR, Johnson TE: Confirmation and fine mapping of ethanol sensitivity quantitative trait loci, and candidate gene testing in the LXS recombinant inbred mice. *J Pharmacol Exp Ther* 2006, 319:299-307.

Bennett B, Carosone-Link P, Beeson M, Gordon L, Phares-Zook N, Johnson TE: Genetic dissection of quantitative trait locus for ethanol sensitivity in long- and short-sleep mice. *Genes Brain Behav* 2008, 7:659-668.

Bennett B, Larson C, Richmond PA, Odell AT, Saba LM, Tabakoff B, Dowell R, Radcliffe RA: Quantitative trait Locus mapping of acute functional tolerance in the LXS recombinant inbred strains. *Alc Clin Exp Res* 2015, 39**:**611-620.

Brunschwig H, Levi L, Ben-David E, Williams RW, Yakir B, Shifman S (2012) **Fine-scale maps of recombination rates and hotspots in the mouse genome.** Genetics 191, 757-64.

Chen K, Wallis JW, McLellan MD, Larson DE, Kalicki JM, Pohl CS, McGrath SD, Wendl MC, Zhang Q, Locke DP, Shi X, Fulton RS, Ley TJ, Wilson RK, Ding L, Mardis ER: **BreakDancer: an algorithm for high-resolution mapping of genomic structural variation.** *Nat Methods* 2009, **6**:677–681.

Darlington TM, Ehringer MA, Larson C, Phang TL, Radcliffe RA (2013) Transcriptome analysis of Inbred Long Sleep and Inbred Short Sleep mice. Genes Brain Behav 12, 263-274

Halper-Stromberg E, Steranka J, Burns KH, Sabunciyan S, Irizarry RA: **Visualization and probability-based scoring of structural variants within repetitive sequences**. *Bioinformatics* 2014, **30**:1514–1521.

Keane TM, Goodstadt L, Danecek P, White MA, Wong K, Yalcin B, Heger A, Agam A, Slater G, Goodson M, Furlotte NA, Eskin E, Nellaker C, Whitley H, Cleak J, Janowitz D, Hernandez-Pliego P, Edwards A, Belgard TG, Oliver PL, McIntyre RE, Bhomra A, Nicod J, Gan X, Yuan W, van der Weyden L, Steward CA, Bala S, Stalker J, Mott R, Durbin R, Jackson IJ, Czechanski A, Guerra-Assuncao JA, Donahue LR, Reinholdt LG, Payseur BA, Ponting CP, Birney E, Flint J, Adams DJ (2011) **Mouse genomic variation and its effect on phenotypes and gene regulation.** Nature 477, 289-294

Killick R, Eckley I: **changepoint: An R Package for changepoint analysis**. *Lancaster Univ* 2013:1–15.

Lander E, Kruglyak L: Genetic dissection of complex traits: guidelines for interpreting and reporting linkage results. *Nat Genet* 1995, 11**:**241-247.

Markel PD, Fulker DW, Bennett B, Corley RP, DeFries JC, Erwin VG, Johnson TE: Quantitative trait loci for ethanol sensitivity in the LS x SS recombinant inbred strains: interval mapping. *Behav Genet* 1996, 26:447-458.

Markel PD, Bennett B, Beeson M, Gordon L, Johnson TE: Confirmation of quantitative trait loci for ethanol sensitivity in long-sleep and short-sleep mice. *Genome Research* 1997, 7:92-99.

McClearn GE, Kakihana R (1981) **Selective breeding for ethanol sensitivity: Short-Sleep and Long-Sleep mice. In Development of Animal Models as Pharmacogenetic Tools**, M.G. E., R.A. Deitrich, V.G. Erwin, eds. Rockville, MD: National Institute on Alcohol Abuse and Alcoholism, pp 147-159

Ramsköld D, Wang ET, Burge CB, Sandberg R: **An abundance of ubiquitously expressed genes revealed by tissue transcriptome sequence data**. *PLoS Comput Biol* 2009, **5**.

Wong K, Keane TM, Stalker J, Adams DJ: **Enhanced structural variant and breakpoint detection using SVMerge by integration of multiple detection methods and local assembly.** *Genome Biol* 2010, **11**:R128.

Supplemental Figures

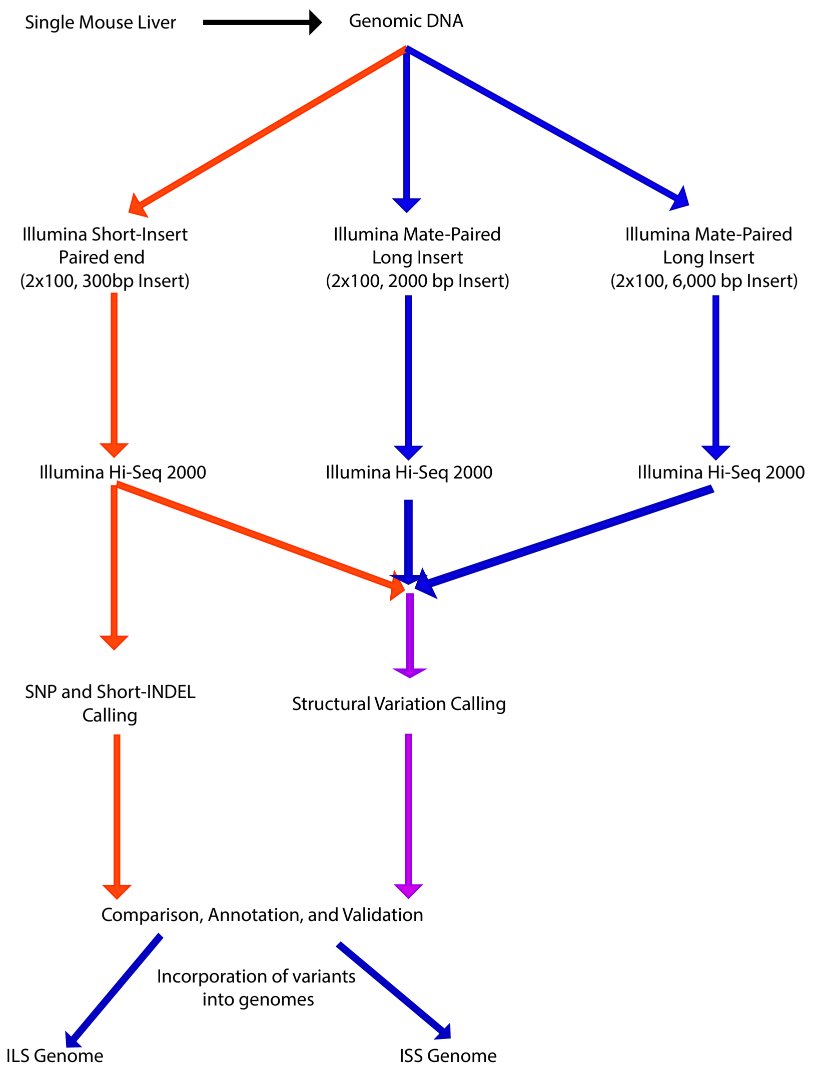

Figure S1: Whole genome sequencing strategy.
Three libraries were prepared and sequenced. Red lines indicate short insert library; blue lines indicate mate-paired libraries; purple lines indicate all library types. We note that large structural variations were not incorporated into our strain specific genomes.

Chromosome 1

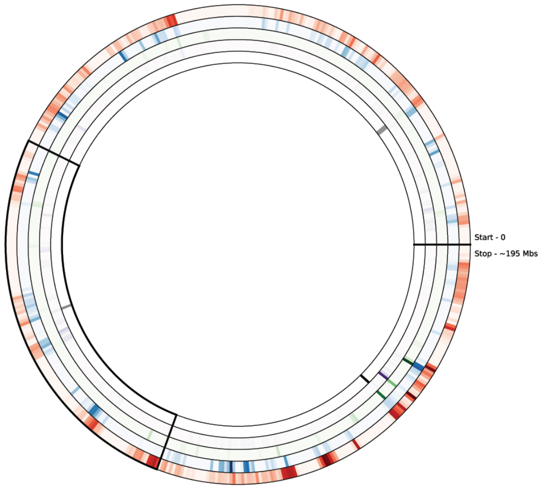

Chromosome 2

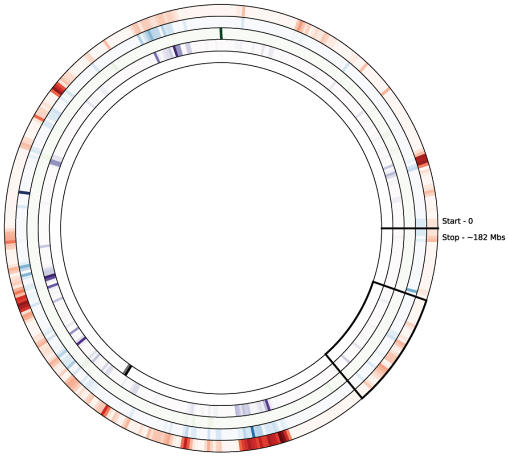

Chromosome 3

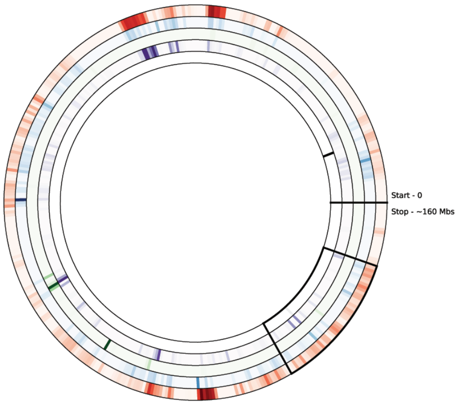

Chromosome 4

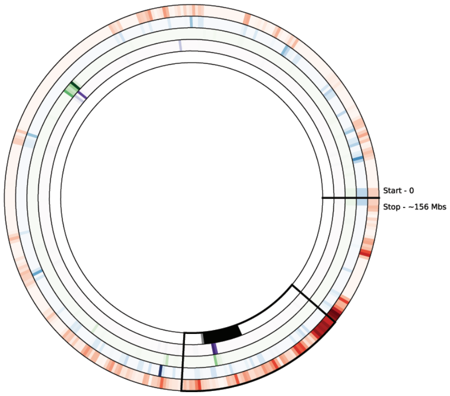

Chromosome 5

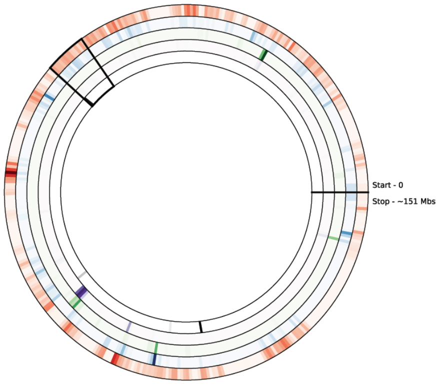

Chromosome 6

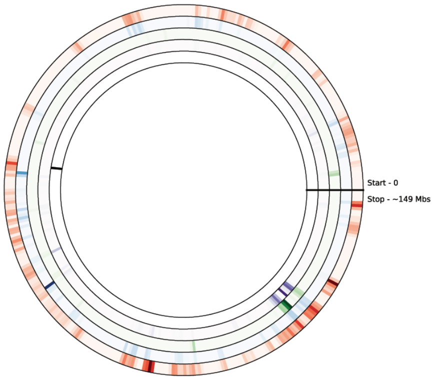

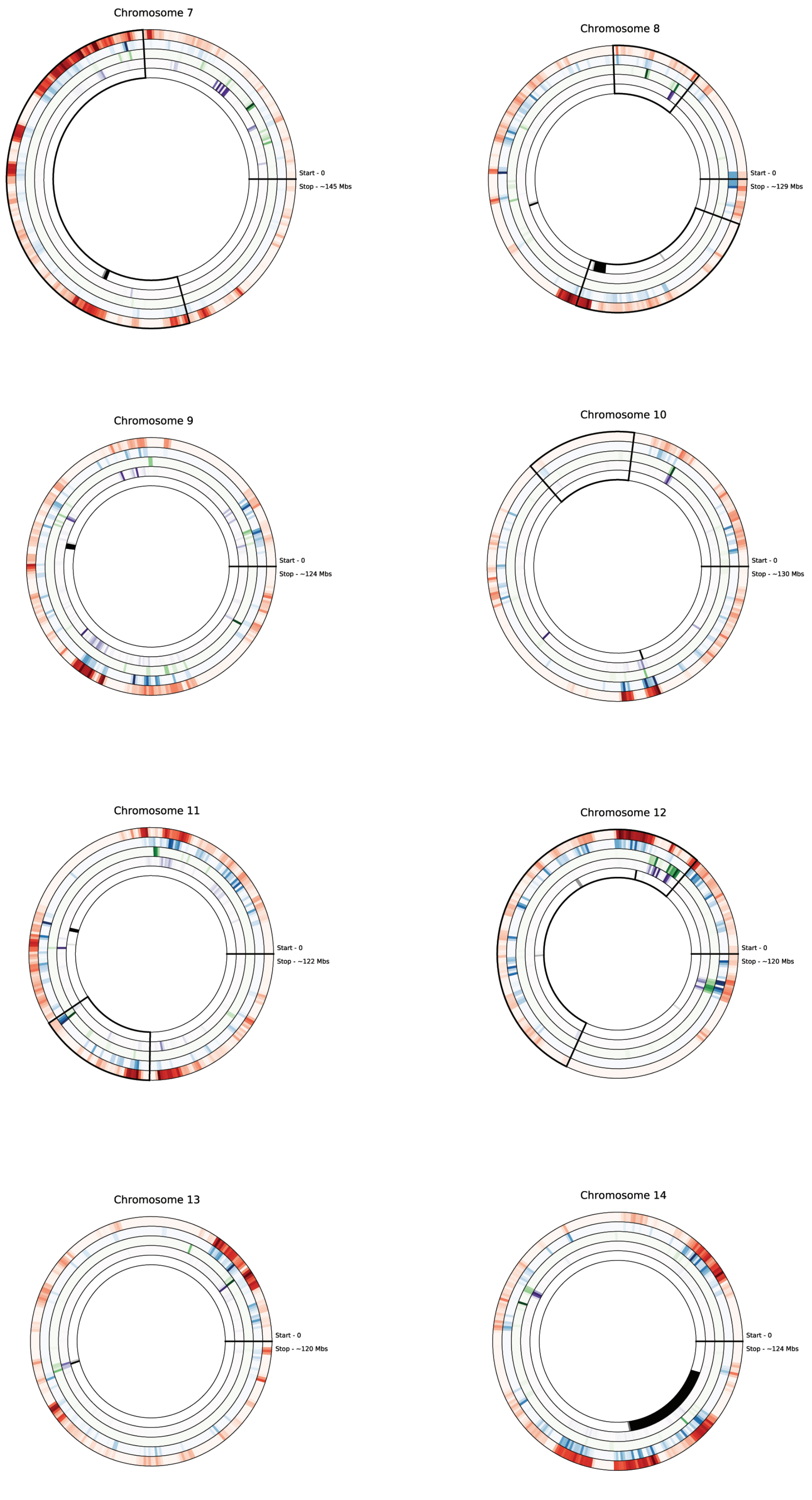
Chromosome 7
Start - 0
Stop - ~145 Mbs
Chromosome 8
Start - 0
Stop - ~129 Mbs
Chromosome 9
Start - 0
Stop - ~124 Mbs
Chromosome 10
Start - 0
Stop - ~130 Mbs
Chromosome 11
Start - 0
Stop - ~122 Mbs
Chromosome 12
Start - 0
Stop - ~120 Mbs
Chromosome 13
Start - 0
Stop - ~120 Mbs
Chromosome 14
Start - 0
Stop - ~124 Mbs

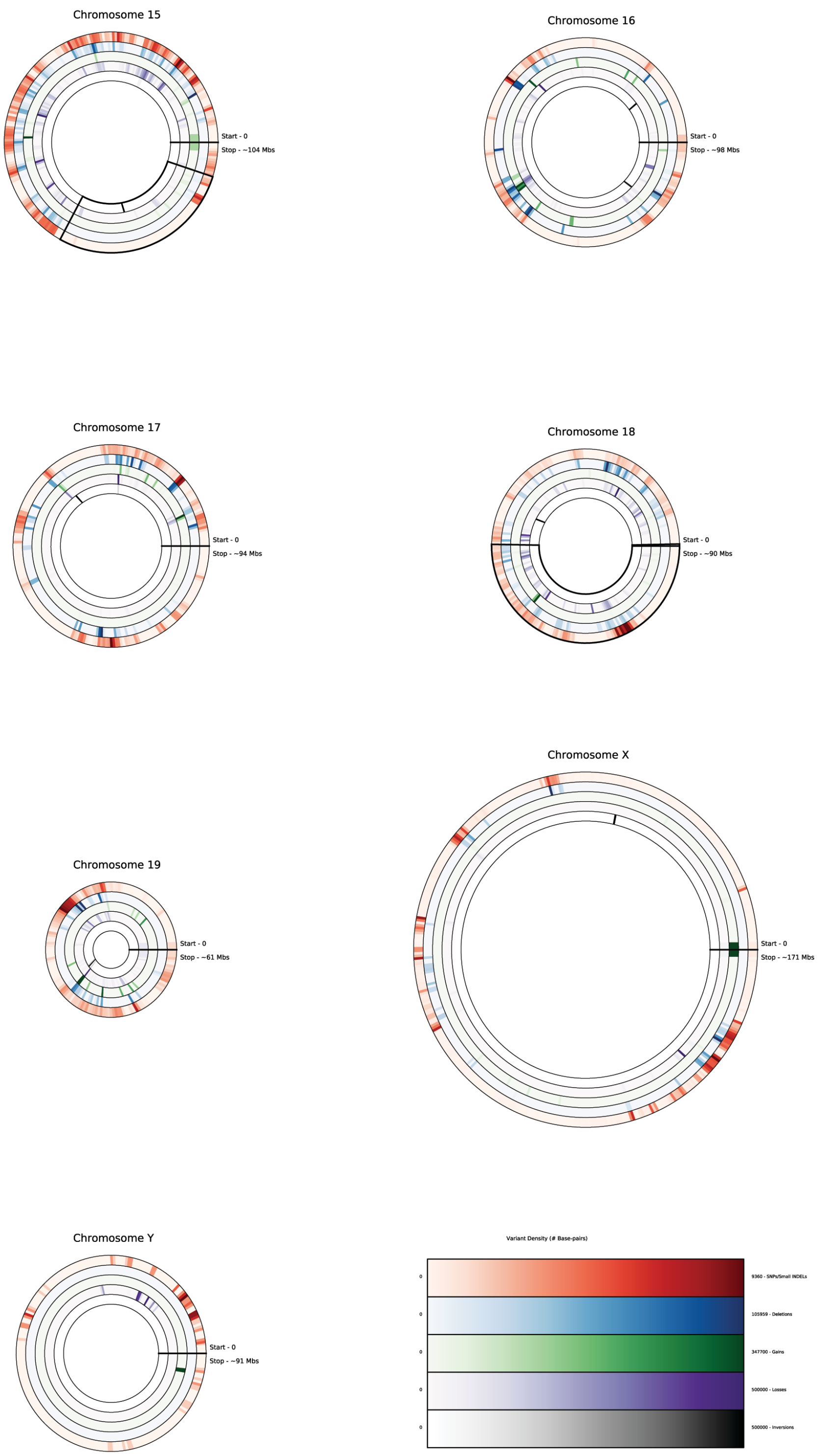

Figure S2: Genome wide distribution of all variants (SNP+indel, SVs)
All variants plotted genome-wide summarized by a sliding window approach (500k windows with 100k stepsize). Concentric circles (from outer to inner): SNPs and small indels, deletions, gains, losses and inversions.

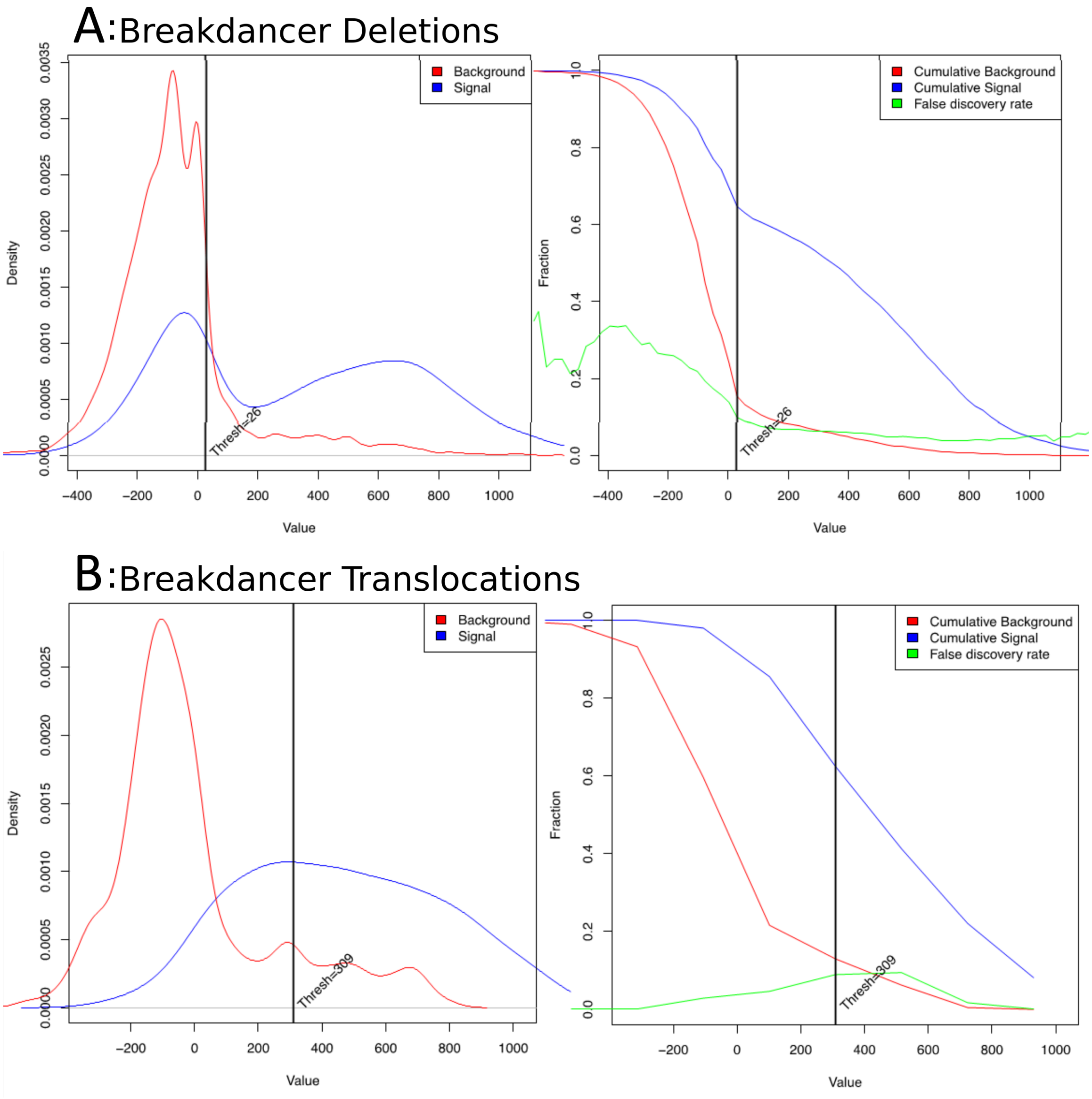
A:Breakdancer Deletions
Background
Signal
Density
Value
Thresh=26
Cumulative Background
Cumulative Signal
False discovery rate
Fraction
B:Breakdancer Translocations
Thresh=309

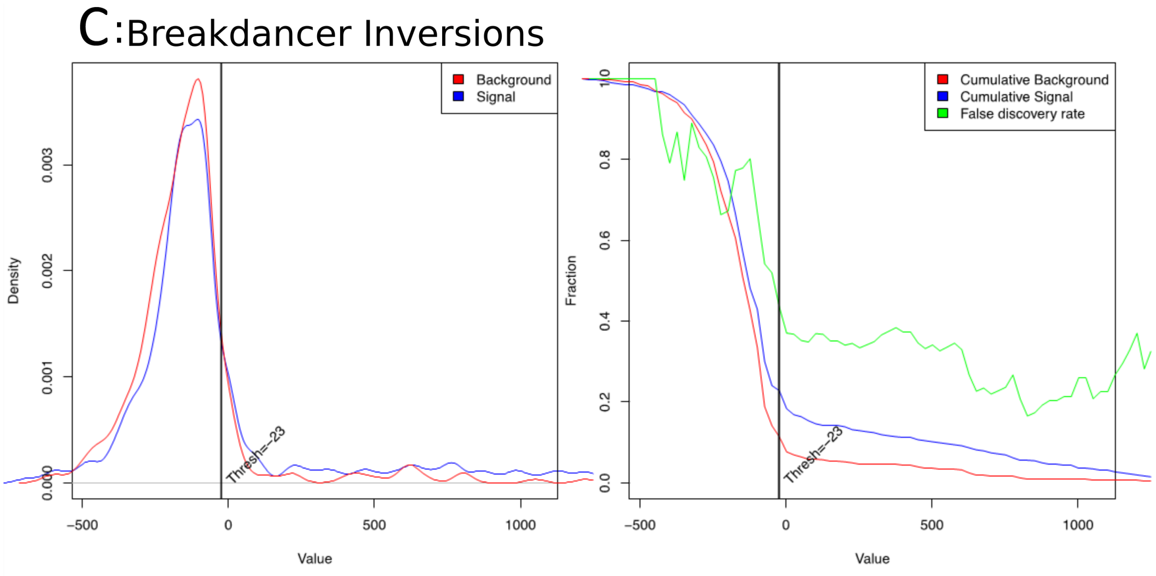
C:Breakdancer Inversions
Background
Signal
Density
Value
Thresh=-23
Cumulative Background
Cumulative Signal
False discovery rate
Fraction
Value
Thresh=-23

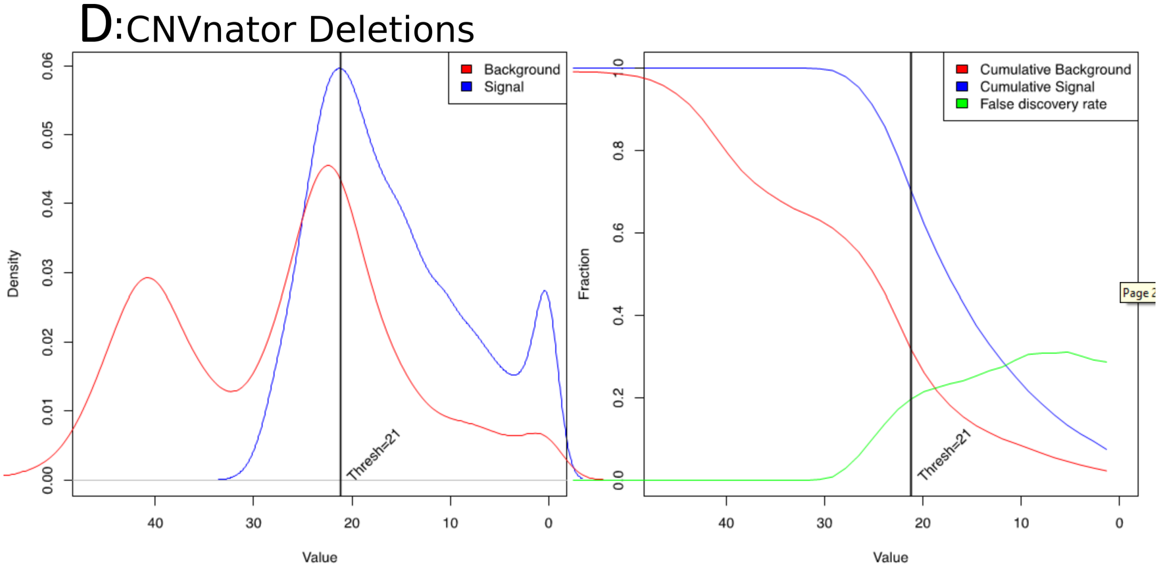
D:CNVnator Deletions
Background
Signal
Density
Value
Thresh=21
Cumulative Background
Cumulative Signal
False discovery rate
Fraction
Value
Thresh=21

E :CNVnator Gains

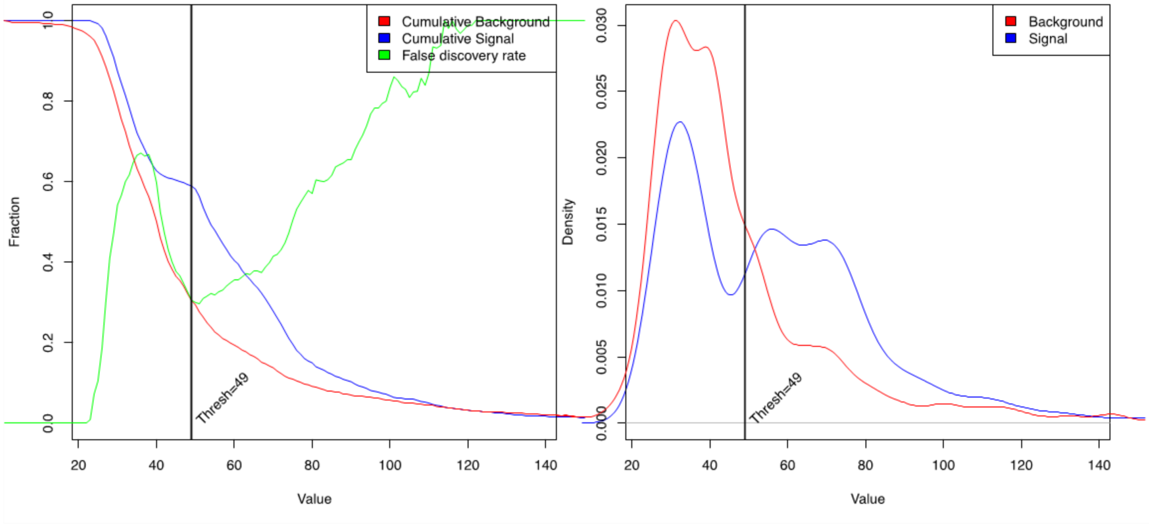

Figure S3: Determining strain specific structural variants.
For each of the 5 methods: Breakdancer (A) Deletions, (B) Translocations, (C) Inversions, CNVnator (D) Deletions and (E) Gains we plot two graphs. In each case, two graphs are presented:
Left: Distribution plots of scores for mouse of origin SV calls (labeled signal, in blue) and corresponding loci tested in the alternate mouse (labeled background in red). Plots were generated using a method developed by Ramskold et al. Ascending scores signify stronger evidence for the event based upon paired-end mapping metrics. The threshold point, indicates the minimum score for assigning an SV as high confidence.
Right: Cumulative distribution plot of scores for mouse of origin SV calls (labeled signal, in blue) and corresponding loci tested in the alternate mouse (labeled background in red) with false discovery rate curve in green. The threshold point, indicates the minimum score for assigning an SV as high confidence. In order to make a strain-specific high confidence call, the score from the mouse of origin event needed to be at the threshold point or above, and the score from the alternate mouse for the same event needed to be less than the threshold point.

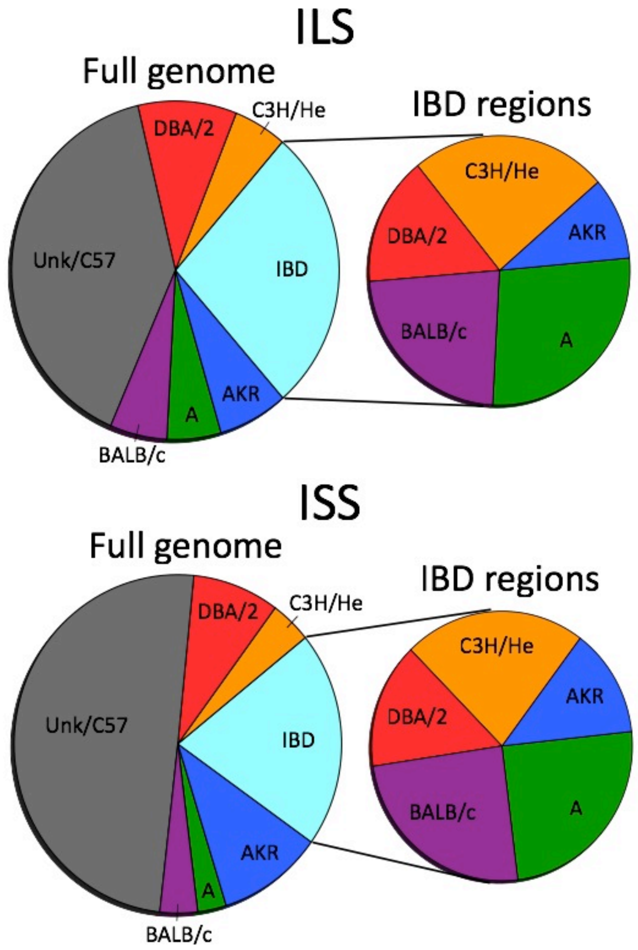

Figure S4: Inferred ancestry of ILS and ISS.
Genome-wide distribution of ancestor assignments based on consistency of ILS and ISS specific variations with ancestor sequence in particular regions. Ancestry is inferred using a hidden Markov model on SNPs and validated by consistency of indels. Regions that are identical by descent between two or more strains are labeled IBD and then subdivided by the possible parental labels.

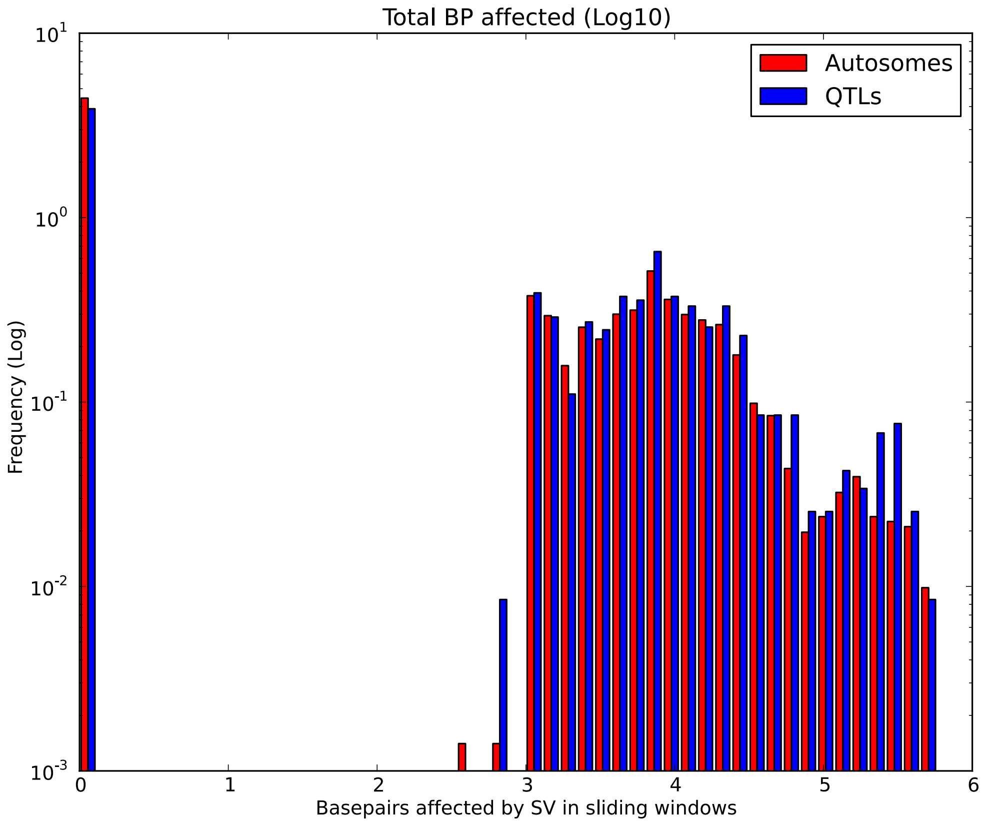

Figure S5: Distribution of structural variants in QTLs.
Histogram summarizing the structural variant density (SVs/kb) throughout the genome on autosomes. Red for non-QTL regions; blue for QTL regions.

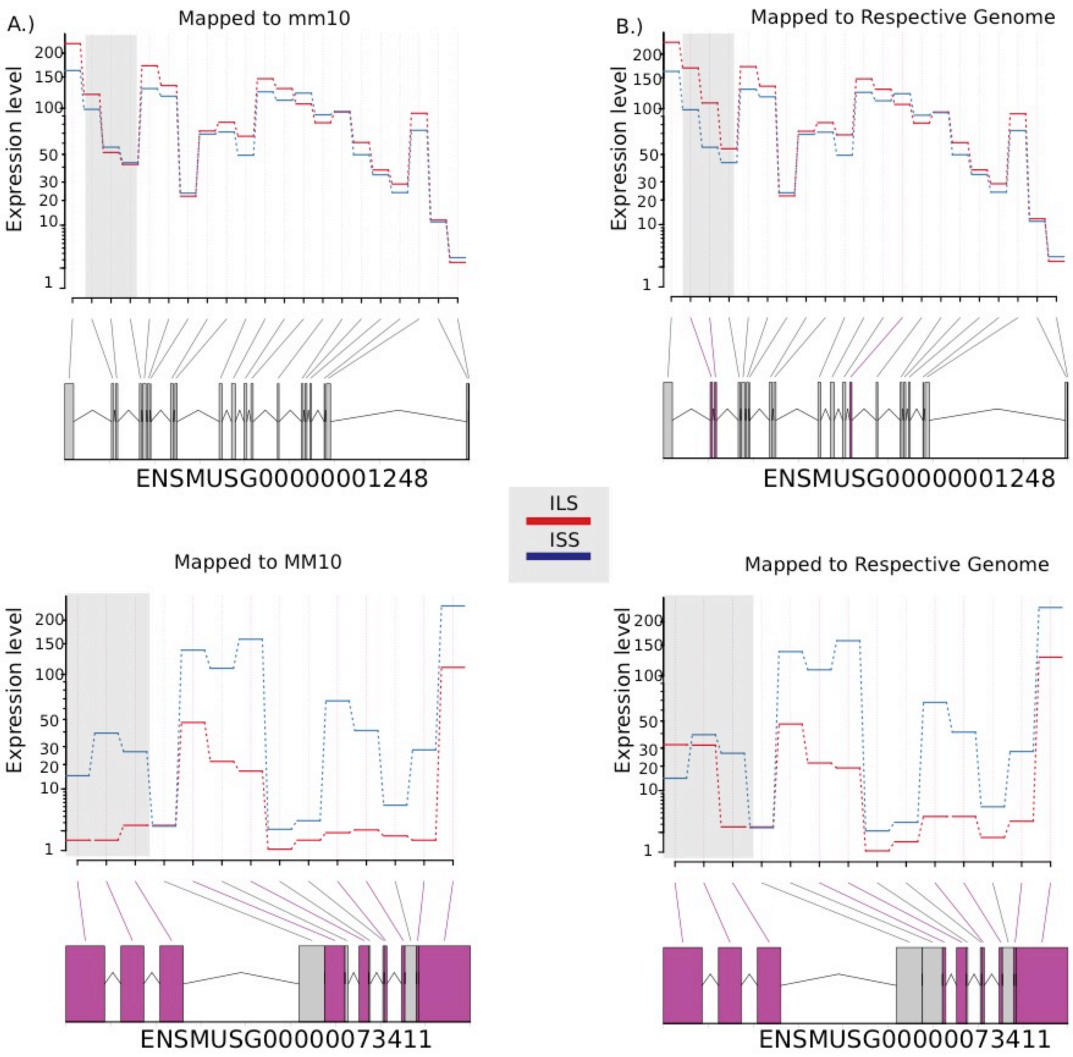

Figure S6: Examples of Impact of genome on Differential expression.
(A) DEXSeq gene plot showing expression level (y-axis) for each exon (x-axis) for ENSMUSG00000001248 in ILS (red) and ISS (blue) samples mapped to mm10 (left) and respective genomes (right). Exonic bins not considered differentially expressed (DE) (gray box) against mm10 become DE when mapped to the respective genomes due to an increase in expression level for the ILS samples. (B) Another example of a DEXSeq gene plot for ENSMUSG00000073411 mapped to mm10 (left) and the respective genome (right). Exons are changing in expression and significance between mm10 and the respective genomes.

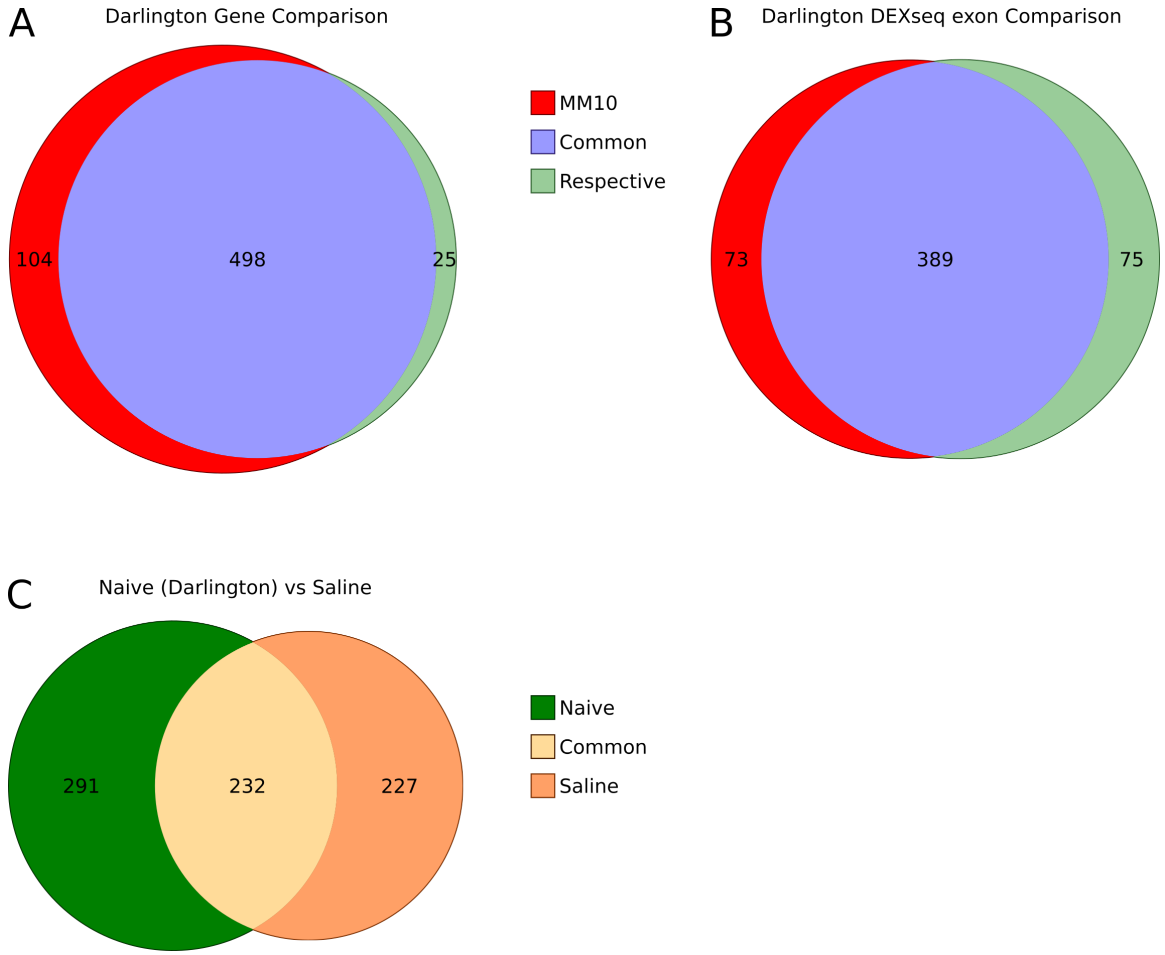

Figure S7: Reanalysis of the Darlington data using the strain specific genomes.
Reads from Darlington et. al. are mapped to mm10 versus the strain-specific genomes (created from SeqNature). Venn diagrams compare the set of (A) differentially expressed genes and (B) DEXSeq-annotated exons. Coloring as in Figure 4. (C) The Darlington et. al. data, which was conducted in naïve mice, is then compared to the saline treated mice of this study using the strain-specific genomes.

SUPPLEMENTAL TABLES

Table S-1: DNA Sequencing Library Statistics for unmapped and unaltered fastq files. R1 and R2 denote 1st and 2nd read in pair, respectively.

Library	Reads Sequenced	Bases Sequenced (non-N)	N-bases Sequenced
**ILS_10kb R1**	58,215,133	5,879,578,116	150,317
**ILS_10kb R2**	58,215,133	5,879,219,063	509,370
**ILS_4kb R1**	23,576,708	2,381,175,614	71,894
**ILS_4kb R2**	23,576,708	2,378,122,646	3,124,862
**ILS_shortInsert R1**	427,367,178	42,706,002,577	30,715,223
**ILS_shortInsert R2**	427,367,178	42,736,092,982	624,818
**ISS_10kb R1**	69,219,229	6,990,962,549	179,580
**ISS_10kb R2**	69,219,229	6,990,539,990	602,139
**ISS_4kb R1**	46,021,645	4,648,045,133	141,012
**ISS_4kb R2**	46,021,645	4,642,087,158	6,098,987
**ISS_shortInsert R1**	439,844,784	43,965,264,033	19,214,367
**ISS_shortInsert R2**	439,844,784	43,973,923,445	10,554,955

Table S-2: Raw fastq file statistics for all ILS and ISS RNA sequencing libraries. R1 and R2 denote 1st and 2nd read in pair, respectively.

**Library**	**Reads Sequenced**	**Bases Sequenced (Non-N)**	**N-Bases Sequenced**
**ILS1_R1**	24,026,433	2,402,591,300	52,000
**ILS1_R2**	24,026,433	2,402,466,505	176,795
**ILS2_R1**	18,913,037	1,891,129,879	173,821
**ILS2_R2**	18,913,037	1,889,521,078	1,782,622
**ILS3_R1**	21,371,370	2,137,083,257	53,743
**ILS3_R2**	21,371,370	2,135,107,890	2,029,110
**ISS1_R1**	48,804,254	4,880,318,697	106,703
**ISS1_R2**	48,804,254	4,880,054,821	370,579
**ISS2_R1**	31,796,992	3,179,400,691	298,509
**ISS2_R2**	31,796,992	3,176,562,877	3,136,323
**ISS3_R1**	46,765,353	4,695,675,914	27,624,739
**ISS3_R2**	46,765,353	4,7158,33,721	7,466,932

Table S-3: Adapter trimmed mate pair DNA library statistics. R1 and R2 denote 1st and 2nd read in pair, respectively.

Library	Reads Sequenced	Bases Sequenced (Non-N)	N-Bases Sequenced
**ILS1_R1_10kb**	49,905,545	4,122,128,767	119,741
**ILS1_R2_10kb**	49,905,545	4,103,875,420	369,447
**ILS2_R1_4kb**	20,298,310	1,689,794,868	57,717
**ILS2_R2_4kb**	20,298,310	1,678,686,881	1,752,727
**ISS1_R1_10kb**	56,993,915	4,500,989,262	134,746
**ISS1_R2_10kb**	56,993,915	4,477,800,377	399,236
**ISS2_R1_4kb**	38,326,474	3,038,507,943	108,228
**ISS2_R2_4kb**	38,326,474	3,019,247,067	2,949,465

Table S-4: Manual examination of why Affymetrix SNPs were missed in DNA sequencing data.

**Reason Missed**	**Frequency**	**Percent of Total Misses**
Low quality mapping issues	16	13.91%
Small insertions/deletions	18	15.65%
Large deletions	38	32.17%
liftOver issues (no mutation in reads)	13	11.30%
False negative	21	17.39%
CNV	3	2.61%
Actually called It, within a few bp	8	6.96%

Table S-5: Score cutoffs for different types of structural variants.

Event Category	Score Threshold	Method	FDR	MOOS Exceeding Threshold	AMS Exceeding Threshold	Strain Specific Calls
Gains	49	Median Coverage	0.33	1597	826	773
Deletions[1]	26	TargetSeqView	0.1	3212	767	2579
Deletions[2]	21	Median Coverage	0.2	6069	2746	3635
Translocations	309	TargetSeqView	0.09	130	27	106
Inversions	-23	TargetSeqView	0.44	92	46	60

Definitions of Abbreviations: MOOS = mouse of origin scores. AMS = alternate mouse scores. FDR: False Discovery Rate

1- Deletions Called by BreakDancer

2- Deletions Called by CNVnator

Table S-6: QTLs in ILS and ISS strains.

Probability [1]	Name [2]	Chrom.	Beg, Mb [3]	End, Mb [3]	Traits [4]	References	Mapping populations	Interval reference [5]
Significant	*Lore1*	1	83.8	135.5	LORR, BEC2_S, LORR_E, LORR_S, BECRR, BEC2_E	1,2,4,6,7,8, unpublished (Radcliffe)	LSxSS_RI, ILSxISS_F2, congenics, LXS_RI	2
Significant	*Lore2*	2	157.1	172.6	BECRR, LORR	1,2,3,4	LSxSS_RI, ILSxISS_F2, congenics	2
Significant	QTL-A	4	116.0	139.1	LORR, RT, AFT_E	1,8	LSxSS_RI, LXS_RI	8
Significant	*Lore3*	8	90.3	122.3	LORR, LORR_S, BECRR	2,6,8	LSxSS_RI, ILSxISS_F2, LXS_RI	2
Significant	*Lore4*	11	72.3	91.3	LORR, BECRR	2,4,7	ILSxISS_F2, congenics	2
Significant	*Lore5*	15	69.8	98.8	LORR, BECRR	2,4,7	ILSxISS_F2, congenics	2
Significant	*Lore6*	18	45.1	91.0	LORR, BECRR	1,6	LSxSS_RI, ILSxISS_F2	2
Suggestive	QTL-B	3	133.4	151.7	LORR	1,6	LSxSS_RI, LXS_RI	6

Suggestive	QTL-C		52.8	58.4	BEC2_E	8	LXS_RI	8
Suggestive	*Lore7*	7	37.7	115.3	LORR, BECRR	1,2	LSxSS_RI, ILSxISS_F2	2
Suggestive	QTL-D	8	18.3	33.1	LORR_E	8	LXS_RI	8
Suggestive	QTL-E	10	30.0	47.9	BEC2_E	Unpublished (Radcliffe)	LXS_RI	Unpublished (Radcliffe)
Suggestive	QTL-F	12	16.3	81.7	LORR, LORR_E, LORR_S	1,8	LSxSS_RI, LXS_RI	8

[1] The QTL was considered either “significant” or “suggestive” by the authors of one or more of the original studies, generally by the criteria of Lander and Kruglyak (1995; ref. 9).
[2] QTL names starting with “*Lore*” were named by the authors of the original study and can be found in the MGI database (http://www.informatics.jax.org/); all others have been named specifically for the current study.
[3] QTL confidence intervals were calculated in the original study and generated using one of several approaches as described in the reference listed in the column “Interval reference”.
[4] LORR: duration of the loss of righting response, naïve mice; LORR_S: duration of the loss of righting response, 24 hours following a saline injection; LORR_E: duration of the loss of righting response, 24 hours following an ethanol injection (5.0 g/kg); BECRR: blood ethanol concentration at the regain of the righting reflex, naïve mice; BEC2_E: blood ethanol concentration at the regain of the righting reflex, 24 hours following an ethanol injection (5.0 g/kg); AFT_E: acute functional tolerance for LORR, 24 hours following an ethanol injection (5.0 g/kg); RT: rapid tolerance for LORR. See references for more information.
[5] Confidence intervals were obtained from the listed reference. Coordinates that were in cM units or from earlier mouse genome builds were converted to GRCm38/mm10 as necessary using the Mouse Map Converter tool (http://cgd.jax.org/mousemapconverter/).

Table S7: Change in read mapping between reference genome (MM10) and strain specific genomes.

	**ILS1**	**ILS2**	**ILS3**	**ISS1**	**ISS2**	**ISS3**
R1 Total	24026433	18913037	21371370	48804254	31796992	46765353
R1 Unmapped (MM10)	5782230	4719005	6091810	14567460	7126285	14450005
R1 Unmapped (Strain Specific)	5573225	4573454	5915797	14163719	6909352	13968690
R1 Mapping	16429782	12634080	13918607	31376180	21541708	29036805

Not Changed						
R1 NonUnique to Unique	6563	5418	5368	8965	6187	7558
R1 Unique to NonUnique	8506	5859	7335	16536	9491	12784
R1 Unmapped to Unique Mapped	210169	146626	177345	405943	218952	483555
R1 Mapped to Unmapped	1501	1420	1607	2720	2404	3238
R1 Unique Moved	13579	11261	12076	19827	13470	17493

	**ILS1**	**ILS2**	**ILS3**	**ISS1**	**ISS2**	**ISS3**
R2 Total	24026433	18913037	21371370	48804254	31796992	46765353
R2 Unmapped (MM10)	7417517	5955724	7310623	17530312	9217655	15255983
R2 Unmapped (Strain Specific)	7114373	5733667	7054465	16964295	8875054	14757671
R2 Mapping Not Changed	14920450	11501751	12784752	28652355	19643973	28258252
R2 NonUnique	5595	4675	4672	7548	5285	7449

to Unique						
R2 Unique to NonUnique	8037	5213	6808	15727	8667	12502
R2 Unmapped to Unique Mapped	304885	223948	258130	568974	345663	501057
R2 Mapped to Unmapped	2223	2366	2358	4079	3819	3705
R2 Unique Moved	14054	11806	12603	20174	14343	20848

	**ILS1**	**ILS2**	**ILS3**	**ISS1**	**ISS2**	**ISS3**
mm10 Genome Unique Alignments	31398132	24173756	26746146	60107598	41237875	57365627
Strain Unique Genome Alignments	31905077	24539565	27173553	61059966	41789581	58333017
